# What are the implications for practice that arise from studies of medication taking? A systematic review of qualitative research

**DOI:** 10.1371/journal.pone.0195076

**Published:** 2018-05-16

**Authors:** Mohammed Ahmed Rashid, Nadia Llanwarne, Natalie Heyns, Fiona Walter, Jonathan Mant

**Affiliations:** 1 UCL Medical School, Royal Free Hospital, Hampstead, London, United Kingdom; 2 Primary Care Unit, Department of Public Health & Primary Care, University of Cambridge, Strangeways Research Laboratory, Cambridge, United Kingdom; Duke University School of Medicine, UNITED STATES

## Abstract

**Background:**

Despite several decades of evidence supporting the benefits of taking medications in various diseases and healthcare settings, a significant proportion of prescribed treatments are not taken. This review sought to synthesise qualitative research exploring experiences of medication taking around the world, and to determine whether there were consistent messages arising from these studies.

**Methods and findings:**

5 databases (MEDLINE, PsycINFO, EMBASE, SCOPUS, CINAHL) were systematically searched to identify published research papers using qualitative methodologies, which explored medication-taking experiences in patients, citizens, carers, relatives and clinicians. Data were extracted independently by at least two clinician reviewers. Implications for practice from individual papers were charted and coded using thematic content analysis. These were then cross-tabulated with research paper categories to explore emergent patterns with particular implications for practice. 192 papers from 34 different countries were included in the review. Implications for practice fitted into 11 categories: increase family involvement, increase clinician involvement, promote personalised management, address practical barriers, provide ongoing support, promote self-management, adopt a patient-centred approach, improve patient education, address system barriers, increase access to non-prescribing clinicians and improve clinician training. These implications for practice were generally evenly spread across research paper categories.

**Conclusions:**

Implications for practice from the published qualitative literature exploring medication-taking are notably consistent across research methods, disease categories and geographical settings. More recent clinical trials of interventions to improve adherence have started to draw on these findings by focussing on improving clinical interactions and involving patients in healthcare decisions. Promoting patient education and self-management have been widely advocated, and improvements at a system level have been frequently cited in studies from developing countries and those relating to communicable diseases. Regardless of the setting, clinicians and policymakers around the world can focus efforts to improve medication-taking by considering a number of consistently emerging findings.

## Background

Although there is often uncertainty from patients about the benefits of medication taking in particular clinical scenarios [1, 2], there is a large body of robust evidence to support their use in a range of diseases and health settings [3, 4]. However, it is also well recognised that medications are frequently not taken as advised and often not taken at all [5]. For example, in cardiovascular disease prevention, about 40% of individuals do not adhere well to prescribed medications, and this poor adherence is associated with high morbidity and mortality [6]. Many interventions have been tested in randomised controlled trials to address this issue. Although there have been notable individual successes with such interventions, the results have overall been disappointing [7].

Researchers from a variety of clinical and non-clinical disciplines have used qualitative research to try to understand the complex decisions relating to medication taking that are made by patients [8], family members [9], carers [10] and health care professionals [11]. These have included studies of particular drug classes, diseases, systems and patient groups. This literature has evolved over the last few decades and has considered populations from all around the world. It has uncovered a variety of important social and behavioural factors that influence medication adherence, including culture, stigma, and ethnicity. There has also been a particular focus on the provision of medications in areas where there are concerns about overmedicalisation, such as opioid overuse [12] and overtreatment of mental illnesses such as depression [13]. Given the complex interplay of biological, psychological and social factors that is likely to be at play when individuals make choices about medication adherence, the in-depth and interpretive nature of qualitative research is well suited to uncover important insights.

Over 10% of total health care spend in many countries, including the US and the UK, is on drugs [14]. In the UK, £15.5 billion was spent on drugs in the NHS in 2014–15, and 57% of this expenditure was in the community [15]. Failure to address non-adherence therefore reflects a costly waste of health care resource in economic, as well as human, terms.

Given the relative lack of success of interventions that have been developed to address non-adherence, it is timely to review the qualitative literature to ascertain what insights these might offer future interventions. We undertook a qualitative synthesis of international research on medication taking and sought to determine whether there were consistent messages from these studies that apply regardless of study context (such as disease; method; and geographical setting).

## Methods

### Literature search and data extraction

Qualitative research can be challenging to search for and identify [16]. It is less well indexed than quantitative research and often catalogued in databases outside of the medical domain [17]. In order to identify papers that used qualitative methodologies to explore the medication-taking experiences of either patients, carers, family members or clinicians, we conducted searches across 5 different databases (MEDLINE, PsycINFO, EMBASE, SCOPUS, CINAHL) from inception to October 2013 and combined this with identification and review of references in papers obtained. An example search strategy is provided in an appendix S1 File. We included all global papers published in English that explored any disease, medication class or demographic group, including those studying medication taking in healthy populations. Editorials and reviews were excluded, as were papers that did not use a qualitative (textual and interpretive) data collection and analysis. All types of qualitative studies, including those linked to observational or experimental studies, were eligible for inclusion. Mixed methods papers were only included if there were substantial textual data that had been subjected to a recognised qualitative analysis method.

All titles and abstracts were screened by one reviewer (MAR) and a subset (10%) were independently screened by a second reviewer (NH), with no discrepancies in selections. Full text papers were reviewed by at least two reviewers (from MAR, NL, NH) to identify inclusions, with discrepancies resolved by discussion. Data were extracted from papers independently by 2 authors (from AR, NL, NH), with differences being resolved through consensus meetings. All 3 reviewers are registered medical doctors working in the English National Health Service.

### Descriptive analysis of included studies

For each paper, data were extracted about data collection method, year of publication, country of origin, primary readership category of the journal (one of three categories: medical, social science and allied health professional) and the impact factor of the journal (from 2014 or the most recently available for those journals out of print). Where data collection included more than one method (for example, both interviews and focus groups), this was classified as ‘combination’.

As the US and UK were the commonest countries of origin, these were kept as distinct domains and the remainder of countries were divided into developed and developing country groups according to the United Nations World Economic Situation and Prospects 2012 [18]. Papers were also classified according to disease group (mental health, communicable diseases, non-communicable diseases, no specific disease), health setting (hospital healthcare, community healthcare, non-healthcare, combination) and whether or not the research or any of its authors had received financial support from the pharmaceutical industry.

These categories were chosen to enable the reviewers to explore any discrepancies in practice recommendations from different study categories. For example, we hypothesised that papers from medical research teams may differ in their suggestions for practice from those led by non-clinical researchers. Similarly, we hoped to test if there were differences according to the types of disease, whether the papers were published in high impact journals, and whether they had been supported by pharmaceutical industry funding or not.

We documented the analysis method reported by authors in each study, and considered the potential for organising this information. However following repeated discussions between the research team, it proved impossible to meaningfully classify the varying reported analytical choices, and so we agreed not to describe this here. Reviewers rated each paper against a modified 7-point CASP rating score with any discrepancies resolved through consensus meeting. This rating was based on a well validated CASP scoring scale for qualitative research [19]. Although this scale contains 10 points, 3 statements (1–3) were not used in this study as the inclusion criteria for this systematic review meant these were inherently true. Papers scoring less than 50% were excluded and scores of all papers were included in the final results table.

A cross tabulation of these extracted research paper categories was performed against the dominant (see below) implications for practice for each paper, using SPSS 22 software. This was used to generate figures that demonstrate the spread of implications for practice across categories. In light of the large number of implications for practice and variable categories, and the small number of overall included papers, significance testing was not appropriate for these data.

### Analysis of implications for practice

Our analysis was inductive, to the extent that it was driven by the data. We did not impose a pre-existing coding framework. Instead, AR and NL developed the codes following stages of data familiarisation. We recorded clinical recommendations verbatim as reported by the studies’ authors, and as we progressed with data collection, we began to observe thematic patterns. We drew up preliminary categories of recommendation, based on our collected themes, and returned to each study in turn to apply our newly devised set of categories. Through re-examination of the data and consensus meetings with all authors, some themes were expanded or collapsed, before reaching our final 11 categories. One might argue that our approach was partially deductive insofar as we focused our attention on ‘clinical implications’ of the included studies. As clinicians grappling with the everyday challenges of prescribing, our aim was to draw insights of clinical value to daily practice.

We adopted a composite analytical strategy. Whilst much of qualitative research relies on implicit quantification, we make our frequency counts transparent to offer a tangible demonstration of the clinical recommendations afforded by the literature synthesis. In such respects, we adopt a content analytical approach [20, 21]. However, we are not counting words or phrases, nor are we using a pre-defined index system [22]. Instead, we are compiling frequencies across the dataset of overarching themes derived from our own interpretive efforts. In this respect, our approach aligns with a ‘thematic analysis’ [23]. Our stance therefore perhaps most closely reflects what Green and Thorogood have termed ‘thematic content analysis’ [24], an approach that acknowledges the considerable overlap between both traditions.

Where papers had implications for practice fitting into more than one category, a dominant one was agreed. For example, if a paper had findings that fitted into several categories, the reviewers reached consensus on a single ‘dominant’ category of recommendation that was given most coverage and emphasis by the study authors.

## Results

### Descriptive analysis of included studies

Database searches produced 2945 papers in total and full text versions of 225 papers were assessed (see PRISMA flowchart, S1 Fig). The 192 papers that were included in the review all used a qualitative research method to study medication-taking experiences of patients, carers, family members or clinicians. They are listed in S1 Table.

The included papers were published evenly across medical (67, 34.8%), allied health professional (61, 31.7%), and social science (64, 33.3%) journals. The studies were conducted across 34 different countries. Well over half the studies were conducted in the developed world (39 UK, 67 US, 52 developed other) but a notable proportion (34, 18%) reported from developing countries. Over two thirds (132, 68.7%) were studies investigating patients’ experiences of medication taking, with the remaining one third drawing on carer or relatives’ experiences (12), clinician experiences (12) or a combination of the three different groups (39). Most studies used interviews for data collection (124, 64.5%), a fifth used focus group methodology (41, 21.4%), and the remainder used a combination of methods (25, 13%), sometimes including observation. One study relied entirely on the analysis of an online media response forum201, but this was an exception and other studies used conventional study designs. Studies using interviews typically used in-depth and semi-structured approaches, although a small number used telephone interviews.

Studies related to medication-taking for non-communicable diseases (81, 42%), communicable diseases (56, 29.2%), and mental health conditions (43, 22.4%). A small number of studies (12, 6.2%) considered medication-taking amongst healthy participants, or patients and carers with no single disease focus. A higher proportion of communicable disease studies arose from the developing world, whereas mental health studies took place predominantly (91%) in the developed world. Those studies taking place in healthcare settings were broadly evenly spread across both hospital (71, 37.0%) and community care (68, 35.4%). A large majority of studies (133, 69.2%) declared no pharma involvement; 36 (18.8%) studies did not mention their source of funding, and 23 (12.0%) studies either declared an affiliation (3, 1.6%) or direct industry funding (20, 10.4%).

The three most frequently reported analytic approaches were content analysis, reference to grounded theory, and thematic analysis. In about? 20% of cases the analytic approach was described in detail, but not attributed to any particular tradition.

### Analysis of clinical implications for practice

A list of the 11 implications for practice categories developed by the reviewers is presented here, with example quotes from papers for illustration.

1 Individualise care plan
  Strategies to improve medication taking should take into account a patient’s individual concerns and issues. This includes all suggestions that call for personalisation of management.

“Medicines information needs to be tailored for the individual”

[25]

2 Address practical barriers for the individual.
  Specific, practical barriers that apply to an individual should be explored and tackled. This includes barriers such as forgetfulness, lack of routine, difficulty obtaining treatments and social isolation. Cost was included in this category but was not frequently cited.

*“Because of taking medication every day*, *I could not find a boyfriend*. *Which man would like to see you take pills every day?”*

[26]

3 Adopt a patient-centred approach
  This refers to clinicians taking a more holistic approach, allowing them to understand the perspective of the individual taking medications, including their ideas, concerns and expectations. This particularly, of course, includes listening to their beliefs about their medications and the rationales for the decisions they make.

*“Clinically*, *this suggests that these representations should be elicited and addressed*, *taking into account the patients' own models of pain.”*

[27]

4 Increase clinician involvement
  Clinicians should spend more time with patients to discuss medications.

*“In summary*, *physicians should actively determine whether patients take their prescribed medication or not by creating a non-judgmental*, *respectful atmosphere where the patients feel comfortable sharing their personal view.”*

[28]

5 Ensure long-term follow up
  The patient should be followed up and seen on more than one occasion to support their medication taking behaviour.

*“Ongoing*, *regular discussion with patients about each of their medicines is required…”*

[29]

6 Promote self-management
  Those taking medications should themselves be encouraged to take a more active role in decision-making and monitoring. It includes, for example, facilitating peer support groups and empowering children to be involved in management discussions as appropriate for their age.

*“Well-structured and coordinated trials stopping medication and measuring outcomes relevant to adolescents*, *parents*, *teachers*, *doctors*, *and/or other stakeholders may help ensure a developmentally appropriate transition from family to self-management of ADHD.”*

[30]

7 Increase family or carer involvement
  Patient experiences of medication-taking would be enhanced if there was greater involvement of family members or carers. Unsurprisingly, this was commonly mentioned in papers that relate to children or older people. The precise role of the family or carer varied and included a greater practical responsibility such as giving reminders and more supportive functions, such as providing advice and counselling.

“Interventions to support and guide parents throughout the decisional process are critical to meet the needs of families of children with ADHD.”

[31]

8 Improve patient education
  Strategies should focus on providing individuals with more information to support them to make decisions about whether or not to take medications. This includes information in a variety of formats, which may be related to medications themselves or underlying disease states that they are being used for.

“It requires health education to emphasise the progressive nature of diabetes and the eventuality of insulin therapy at an early stage of the illness.”

[32]

9 Address system barriers
  Medication-taking could best be optimised by making changes at the health system broadly. This may include improvements to the healthcare setting but may also include broader suggestions including overcoming geographical and financial barriers.

*“Optimizing adherence may require that antiretroviral therapy programs be linked to other services*, *including drug addiction treatment*, *mental health services and vocational treatment and support.”*

[33]

10 Increase access to non-prescribing clinicians
  Individuals should have access to a healthcare professional other than the prescriber (usually a physician). This includes pharmacists, nurses and other healthcare worker roles.

*“…the clinical nurse specialist is paramount in assisting both younger and older renal transplant recipients with immunosuppressive medication taking and*, *consequently*, *in fostering better outcomes.”*

[34]

11 Improve staff training
  Healthcare staff involved in medication provision need training to improve their ability to support those taking treatments. It includes a variety of improvement areas such as prescribing guidelines, counselling about treatments options and communication skills.

“…training doctors and nurses in effective prevention and management of non-adherence.”

[35]

Few of the publication characteristics seemed to relate to the prevalence of individual implications for practice. An even spread of implications for practice were found across the dataset (see S2 Fig, panel A); individualised care planning, addressing practical barriers for the individual, patient-centredness and increasing clinician involvement were the most common.

Papers published across all social science, clinical and allied health professional journals made implications for practice pertaining to all 11 implications for practice (S2 Fig, panel B). Papers in the clinical journals seemed to have slightly more emphasis on optimising clinician involvement, whilst papers in the allied health journals more frequently recommended improving access to non-prescribing clinicians. System level barriers were more commonly referred to in papers published in the social science journals.

Looking at the spread of implications for practice across disease categories (S2 Fig, panel B), the most striking feature is the higher proportion of studies calling for system level changes amongst papers investigating medication-taking of people with communicable diseases, compared to the three other disease categories. Similarly, there were a higher proportion of system level changes in developing countries, compared to developed countries. Implications for practice did not differ according to year of publication or the type of healthcare setting. Considering sources of research funding, studies which reported pharma funding or affiliation contained a higher proportion of implications for practice relating to individual barriers and individual approaches, and did not report on system factors nor on family or carer involvement (S2 Fig, panel B).

S2 Fig—Panel A: Spread of 11 implications for practice across 192 papers on medication-taking experience. Implications for practice are organised according to whether they apply at the clinician or policy level. Panel B: Spread of implications for practice across 192 papers according to journal category (medical, social science, allied health professional AHP); according to disease category (communicable, non-communicable, mental health, no specific disease); and according to pharmaceutical funding status (pharmaceutical funding or affiliation, no pharmaceutical involvement, no funding mention)

## Discussion

This review synthesises qualitative research from around the world that explores medication-taking across all populations and disease groups. Of note, despite the variations in research methods, geography and disease type, the implications for practice across papers were remarkably similar and fit neatly into 11 categories.

The strengths of this review include the systematic search strategy and the use of both clinical and non-clinical databases. Rigor was enhanced by ensuring papers were read by two authors independently with consensus meetings to discuss any divergent results. The diversity of terms used for qualitative data analytic approaches meant no meaningful interpretation of this was possible as distinct categories did not emerge from included papers. Due to the large number of papers, a more detailed analysis of themes using an interpretive methodology such as meta-ethnography was also not possible. Indeed, all reviewers involved in this study are trained clinicians and the absence of additional social science reviewers may have influenced the approach taken. However, the review team has had extensive training and expertise in qualitative methods and indeed, the focus on implications for practice rather than theoretical themes enabled a more applied series of overarching findings which we hope will be useful to practising healthcare professionals. The inclusion of a large number of studies presents a comprehensive overview of the literature on this subject. Moreover, detailed reviews with narrower focus on individual diseases and populations already exist [36, 37, 38].

We included studies published up until October 2013, the time at which the search was conducted. We analysed our data to look for secular trends. We noted that the number of studies researching medication-taking increased significantly after 2006 (>10/year). 2005 marks the beginning of research conducted in the developing world on this subject, and in accordance with this, the practice recommendation to address system factors became more prominent at this time. Between 2006 and 2013 no new themes relating to practice recommendation emerged, and across this period, the relative spread of recommendations remains consistent. We achieved saturation of our themes across the dataset studied, namely each of the eleven themes was identified in at least seven studies. As such, we thought it unlikely that updating the search would give rise to new themes.

There has been interest in medication choices for several decades [39] and observational studies have demonstrated that factors such as low socio-economic status [40], poor social support [41] and depression [42] are all associated with lower adherence to prescribed treatments. As demonstrated in this review, there has also been much qualitative research that seeks to understand these choices. Pound and colleagues (2005) synthesised 37 qualitative studies of lay experiences of medication taking [43], finding that the main reason that people resist medications is an intrinsic preference to avoid them. Their implications for practice closely match those found in this review, including increasing clinician involvement and training although they also suggest there should be an additional policy focus on improving medication safety and tolerability.

There have also been a number of systematic reviews of qualitative research on medication taking that have focussed on individual diseases, including anorexia [36], coronary artery disease [37] and hypertension [38], all finding as this review did that holistic clinician practice and patient education are key strategies. Other studies using this type of methodology have synthesised findings about individual treatments, including TB treatment [44], antidepressants [13], psychotropic medications [45] and even complementary therapies for cancer [46]. Again, implications for practice broadly support those found in this review, including personalising management plans, shared decision making, patient centredness and involving family and carers.

A number of interventions to improve experiences of taking medications recommended in this review have also been tested experimentally. For example, a systematic review and meta-analysis showed mHealth interventions that use mobile phones to aid communication about medications were largely positive, although further, higher quality studies are needed [47]. Similarly, recent systematic reviews of randomised controlled trials have demonstrated that packaging interventions as practical reminders [48] and healthcare provider targeted interventions [49] both seem effective methods of improving medication adherence rates. An updated Cochrane review of randomised controlled trials testing interventions to improve adherence found that the majority of interventions that have been tested have proven ineffective [7]. However, in the more recent studies included in this review, interventions were more complex, multifaceted and involving allied health professionals such as nurses and pharmacists. These more recent studies also had a greater focus on interactions with patients. This suggests that researchers designing interventions to improve adherence are increasingly utilising research findings from the qualitative literature.

Across diverse populations and disease groups around the world, there is notable consistency with regard to implications for practice arising from qualitative research into medication taking experience. For policymakers, there are implications with regard to improving infrastructure and access to healthcare, particularly in communicable diseases and in developing countries. For clinicians, key themes include promoting self-management; considering patient education resources; improving training across all of the healthcare team; and taking a proactive and holistic approach when supporting individuals taking medications. In particular, the qualitative literature brings out the importance of understanding individual perspectives and barriers and using this information to personalise management strategies. These findings fit with the widespread recognition that person-centred care [50] and shared decision-making [51] are important tenets of improving healthcare provision globally. Finally, the availability of ongoing and continuous support seems to be particularly important and this fits with an ongoing focus on maximising continuity of care [52].

Despite the large number of clinical trials that have tested interventions to improve patient adherence, current methods are largely ineffective. The most recent trials of such interventions have tackled issues that are highlighted in this review and in light of the consistency of important themes demonstrated here, further trials should continue to design interventions that focus on these key areas. Although no secular trends were noted in this study, the fast-changing landscape of clinical care means that further research exploring the change in clinical practice recommendations arising from qualitative research is warranted. Given that this review demonstrates the importance of clinicians spending more time with patients, further research on the optimal way to use this time to clarify patients’ values and elicit their preferences is also warranted, building on existing work in this field [53].

As demonstrated in S1 Fig, most papers included in this review contained recommendations that target at clinician level rather than at policy level. Indeed, recommendations such as individualising care and taking a patient-centred approach were amongst the most commonly occurring categories. This suggests that relationships with healthcare professionals are a key influence on patient decisions about whether to take medications and reinforces the importance of shared decision making as a means to overcome medication bias and overmedicalisation. From a policy perspective, ensuring clinicians have the time and skills to engage in these important discussions with patients should be a priority.

This qualitative review has identified consistent issues arising about medication taking across disease groups that should be considered by all clinicians who prescribe drugs in order to minimise the clinical and economic harms that arise from medication non-adherence.

## Supporting information

S1 File Search strategy. (DOCX)

S2 File PRISMA checklist. (DOC)

S1 Fig PRISMA flowchart. (TIF)

S2 Fig Findings—Panel A and Panel B. (TIF)

S1 Table List of included papers. (DOCX)
