# Supplementary material for: What are the implications for practice that arise from studies of medication taking? A systematic review of qualitative research
Source: PLoS One. 2018 May 16;13(5):e0195076. doi: 10.1371/journal.pone.0195076 (PMC5955529; doi:10.1371/journal.pone.0195076)
Supplement: S1 File — (DOCX) [file pone.0195076.s001.docx]

**Appendix 1 – Search terms for MEDLINE Database Search**

1 Medication Adherence/

2 medication.mp.

3 adherence.mp.

4 Patient Compliance/

5 concordance.mp.

6 persistence.mp.

7 Self Administration/

8 drug taking.mp.

9 compliance.mp.

10 Compliance/

11 qualitative.mp.

12 Qualitative Research/

13 phenomen*.mp.

14 ethnograph*.mp.

15 Focus Groups/

16 focus group*.mp.

17 Interview/

18 interview.mp.

19 narrative.mp.

20 thematic.mp.

21 Grounded Theory/

22 grounded theor*.mp.

23 1 or 2 or 3 or 4 or 5 or 6 or 7 or 8 or 9 or 10

24 11 or 12 or 13 or 14 or 15 or 16 or 17 or 18 or 19 or 20 or 21 or 22

25 23 and 24
