# Supplementary figures and images for: What are the implications for practice that arise from studies of medication taking? A systematic review of qualitative research
Source: PLoS One. 2018 May 16;13(5):e0195076. doi: 10.1371/journal.pone.0195076 (PMC5955529; doi:10.1371/journal.pone.0195076)

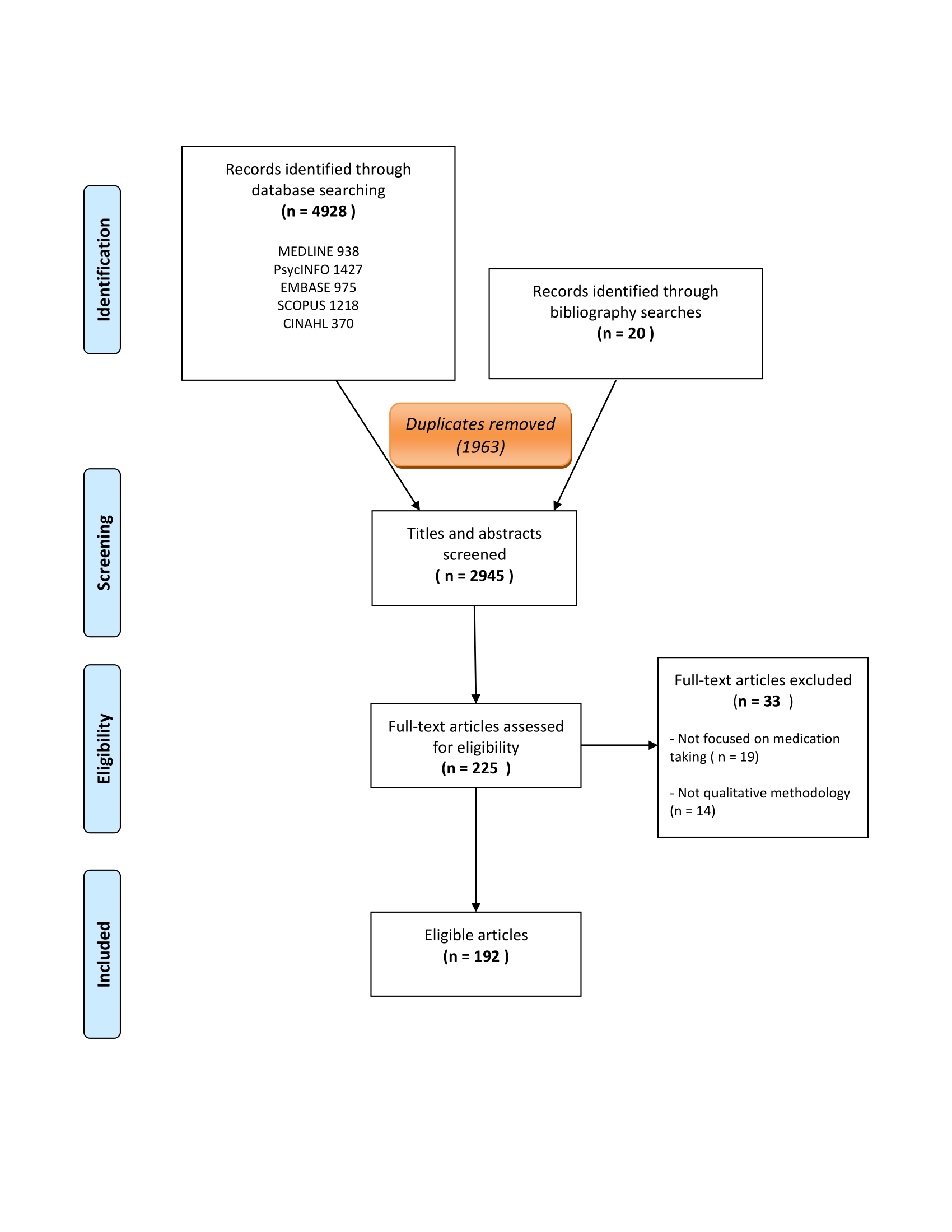

Supplement: S1 Fig — (TIF) [file pone.0195076.s003.tif]

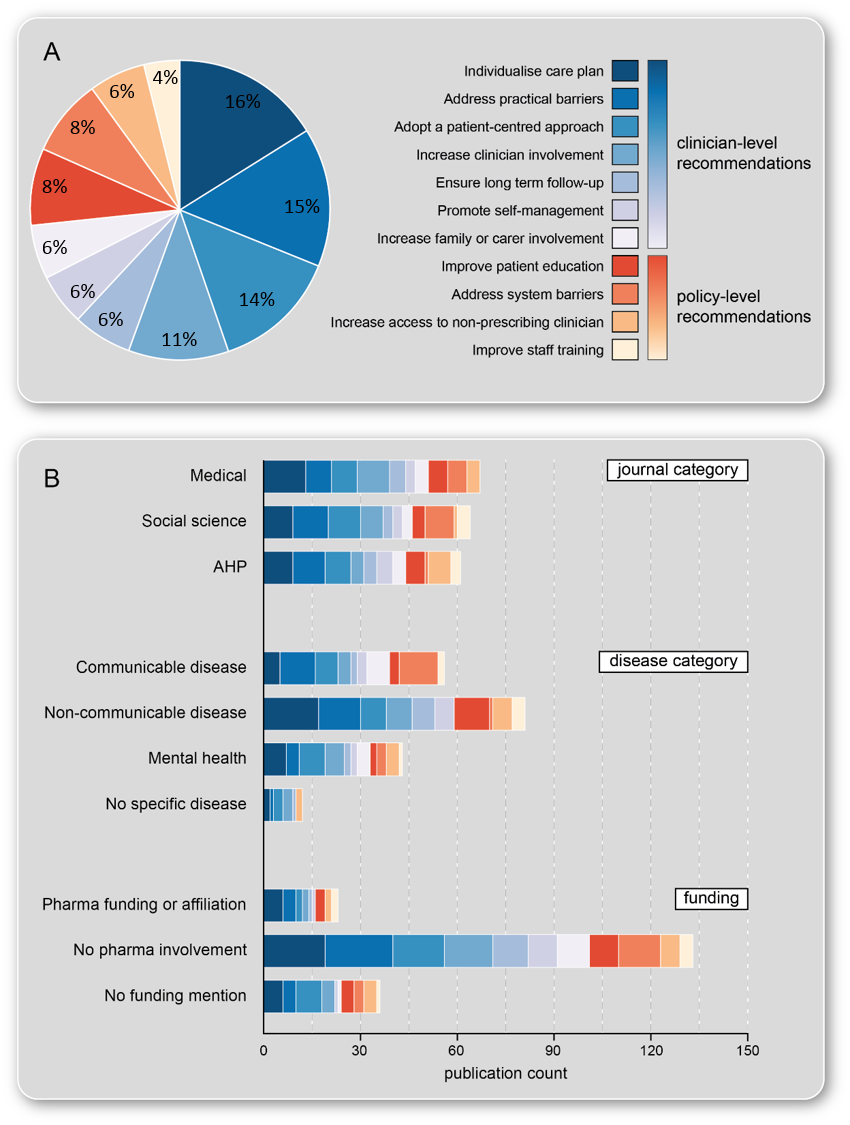

Supplement: S2 Fig — (TIF) [file pone.0195076.s004.tif]
