# Supplementary material for: What are the implications for practice that arise from studies of medication taking? A systematic review of qualitative research
Source: PLoS One. 2018 May 16;13(5):e0195076. doi: 10.1371/journal.pone.0195076 (PMC5955529; doi:10.1371/journal.pone.0195076)
Supplement: S1 Table — (DOCX) [file pone.0195076.s005.docx]

| **Author** | **Year** | **Title** | **Journal category** | **Journal Impact Factor (2014 or most recent)** | **Country category** | **Population** | **Disease group category** | **Health setting** | **Pharma funding** | **Final CASP score** | **Data collection** | **Dominant Recommendation** |
| --- | --- | --- | --- | --- | --- | --- | --- | --- | --- | --- | --- | --- |
| Morgan [1] | 1988 | Managing hypertension: beliefs and responses to medication among cultural groups | Social science | 1.88 | UK | patient | non-communicable disease | community healthcare | no pharma involvement | 5 or less | interviews | R3 |
| Karp [2] | 1993 | Taking anti-depressant medications: resistance, trial commitment, conversion, disenchantment | Social science | 0.78 | US | patient | mental health | community healthcare | no funding mention | 5 or less | interviews | R7 |
| Dowell [3] | 1997 | A qualitative study of medication-taking behaviour in primary care | Medical | 0.74 | UK | patient | no specific disease | community healthcare | no pharma involvement | 6 | interviews | R7 |
| Adams [4] | 1997 | Medication, chronic illness and identity: the perspective of people with asthma | Social science | 2.56 | UK | patient | non-communicable disease | community healthcare | no pharma involvement | 5 or less | interviews | R3 |
| Boath [5] | 1997 | The rise and rise of proton pump inhibitor drugs: patients' perspectives | Social science | 2.56 | UK | patient | non-communicable disease | community healthcare | no funding mention | 5 or less | interviews | R2 |
| Lisper [6] | 1997 | Medicated hypertensive patients' views and experience of information and communication concerning anti-hypertensive drugs | Social science | 2.6 | Developed | patient | non-communicable disease | community healthcare | no pharma involvement | 6 | interviews | R7 |
| Johnson [7] | 1999 | Adherent and nonadherent medication-taking in elderly hypertensive patients | AHP | 1.23 | US | patient | non-communicable disease | hospital healthcare | no pharma involvement | 6 | interviews | R8 |
| Chen [8] | 2000 | ‘Strong medicine': an analysis of pharmacist consultations in primary care | Medical | 0.74 | UK | combination | no specific disease | community healthcare | no pharma involvement | 5 or less | combination | R10 |
| Boyle [9] | 2000 | Medication compliance in older individuals with depression: gaining the views of family carers | AHP | 0.98 | UK | carer/relative | mental health | non healthcare | no funding mention | 6 | focus groups | R1 |
| Francis [10] | 2000 | Caring for people with schizophrenia: family carers' involvement with medication | AHP |  | UK | carer/relative | mental health | community healthcare | no pharma involvement | 6 | interviews | R1 |
| Ivers [11] | 2000 | Decision making in migraine patients taking sumatriptan: an exploratory study | Medical | 3.28 | Developed | patient | non-communicable disease | hospital healthcare | industry funding | 5 or less | interviews | R3 |
| Svensson [12] | 2000 | Reasons for adherence with antihypertensive medication | Medical | 6.18 | Developed | patient | non-communicable disease | combination | no funding mention | 7 | interviews | R7 |
| Usher [13] | 2001 | Taking neuroleptic medications as the treatment for schizophrenia: A phenomenological study | AHP |  | Developed | patient | mental health | non healthcare | no pharma involvement | 6 | interviews | R7 |
| Angermeyer [14] | 2001 | Patients' and relatives' assessment of clozapine treatment | Social science | 5.43 | Developed | combination | mental health | hospital healthcare | no funding mention | 5 or less | interviews | R3 |
| Golin [15] | 2002 | Secret pills: HIV-positive patients' experiences taking antiretroviral therapy in North Carolina | Medical | 1.51 | US | patient | communicable disease | hospital healthcare | no pharma involvement | **7** | focus groups | R4 |
| Happell [16] | 2002 | The role of the inpatient mental health nurse in facilitating patient adherence to medication regimes | AHP | 2.01 | Developed | clinician | mental health | hospital healthcare | industry funding | 6 | focus groups | R10 |
| Wilson [17] | 2002 | Reconciling incompatibilities: A grounded theory of HIV medication adherence and symptom management | Social science | 2.19 | US | patient | communicable disease | combination | no pharma involvement | 5 or less | interviews | R7 |
| Benson [18] | 2002 | Patients' decision about whether or not to take antihypertensive drugs: a qualitative study | Medical | 16.3 | UK | patient | non-communicable disease | community healthcare | no pharma involvement | 6 | interviews | R3 |
| Knudsen [19] | 2002 | Perceptions of young women using SSRI antidepressants: a reclassification of stigma | AHP |  | Developed | patient | mental health | community healthcare | no funding mention | 6 | interviews | R7 |
| Jones [20] | 2002 | The other side of the pill bottle: the lived experience of HIV-positive nurses on HIV combination drug therapy | AHP | 1.23 | US | patient | communicable disease | non healthcare | no pharma involvement | 6 | interviews | R6 |
| Sankar [21] | 2002 | Adherence discourse among African-American women taking HAART | Social science | 2.19 | US | patient | communicable disease | hospital healthcare | no funding mention | 7 | interviews | R4 |
| Karner [22] | 2002 | Conceptions on treatment and lifestyle in patients with coronary heart disease - a phenomenographic analysis | Social Science | 2.6 | Developed | patient | non-communicable disease | hospital healthcare | no pharma involvement | 7 | interviews | R11 |
| Garfield [23] | 2003 | The paradoxical role of antidepressant medication - returning to normal functioning while losing the sense of being normal | AHP | 1.4 | UK | patient | mental health | community healthcare | no pharma involvement | 6 | interviews | R2 |
| Brackis-Cott [24] | 2003 | Pediatric HIV medication adherence: the views of medical providers from two primary care programs | AHP | 1.97 | US | clinician | communicable disease | community healthcare | no pharma involvement | 6 | interviews | R4 |
| Russell [25] | 2003 | Medication-taking beliefs of adult renal transplant recipients | AHP | 0.9 | US | patient | non-communicable disease | hospital healthcare | no pharma involvement | 6 | interviews | R10 |
| Unson [26] | 2003 | Nonadherence and osteoporosis treatment preferences of older women: A qualitative study | Medical | 1.9 | US | patient | non-communicable disease | community healthcare | no pharma involvement | 5 or less | focus groups | R4 |
| Lukoschek [27] | 2003 | African Americans' beliefs and attitudes regarding hypertesnion and its treatment: a qualitative study | Social science | 1.1 | US | patient | non-communicable disease | hospital healthcare | no pharma involvement | 6 | focus groups | R11 |
| Remien [28] | 2003 | Adherence to medication treatment: a qualitative study of facilitators and barriers among a diverse sample of HIV+ men and women in four US Cities | Social science | 3.72 | US | patient | communicable disease | combination | no pharma involvement | 5 or less | interviews | R7 |
| Knudsen [29] | 2003 | Leading ordinary lives: a qulatiative study of young womens' perceived function of antidepressants | AHP | 1.35 | Developed | patient | mental health | community healthcare | no funding mention | 6 | interviews | R7 |
| Carrick [30] | 2004 | The quest for well-being: A qualitative study of the experience of taking antipsychotic medication | Social science | 1.82 | UK | patient | mental health | hospital healthcare | no pharma involvement | **6** | combination | R7 |
| Gascon [31] | 2004 | Why antihypertensive patients do not comply with the treatment: Results from a qualitative study | Medical | 0.74 | Developed | patient | non-communicable disease | community healthcare | no pharma involvement | 6 | focus groups | R2 |
| Happell [32] | 2004 | Wanting to be heard: mental health consumers' experiences of information about medication | AHP | 2.01 | Developed | patient | mental health | non healthcare | no funding mention | 7 | focus groups | R7 |
| Haslam [33] | 2004 | Patients' experiences of medication for anxiety and depression: effects on working life | Medical | 0.74 | UK | combination | mental health | non healthcare | no pharma involvement | 5 or less | focus groups | R5 |
| Wood [34] | 2004 | Medication adherence for HIV positive women caring for children: in their own words | Social science | 2.19 | US | patient | communicable disease | non healthcare | no pharma involvement | 5 or less | interviews | R1 |
| Penza-Clyve [35] | 2004 | Why don't children take their asthma medications? A qualitative analysis of children's perspectives on adherence | Medical | 1.83 | US | patient | non-communicable disease | combination | industry funding | 5 or less | focus groups | R4 |
| Bollini [36] | 2004 | Understanding treatment adherence in affective disorders: a qualitative study | AHP | 0.98 | Developed | combination | mental health | community healthcare | industry funding | 5 or less | focus groups | R11 |
| Deegan [37] | 2005 | The importance of personal medicine: A qualitative study | Medical | 3.12 | US | patient | mental health | community healthcare | no funding mention | 6 | interviews | R7 |
| Connell [38] | 2005 | Strategies to manage hypertension: a qualitative study with black Caribbean patients | Medical | 2.36 | UK | patient | non-communicable disease | community healthcare | no pharma involvement | 6 | interviews | R3 |
| Halkitis [39] | 2005 | The physical, emotional and interpersonal impact of HAART: Exploring the realities of HIV seropositivfe individuals on combination therapy | Social science | 1.88 | US | patient | communicable disease | community healthcare | no funding mention | 5 or less | focus groups | R3 |
| Wrubel [40] | 2005 | Paediatric adherence: perspectives of mothers of children with HIV | Social science | 2.56 | US | carer/relative | communicable disease | combination | no pharma involvement | 6 | interviews | R1 |
| Albus [41] | 2005 | Preferences regarding medical and spychological support in HIV-infected patients | Social science | 2.6 | Developed | patient | communicable disease | hospital healthcare | no pharma involvement | 6 | interviews | R2 |
| Morgan [42] | 2005 | Barriers to uptake and adherence with malaria prophylaxis by the African community in London, England | Social science | 1.28 | UK | patient | communicable disease | non healthcare | no pharma involvement | 6 | focus groups | R9 |
| Kumarasamy [43] | 2005 | Barriers and facilitators to antiretroviral medication adherence amonth patients with HIV in Chenai, India: a qualitative study | Medical | 3.5 | Developing | patient | communicable disease | community healthcare | no pharma involvement | 6 | interviews | R9 |
| Badger [44] | 2006 | Concordance with antidepressant medication in primary care | AHP |  | UK | patient | mental health | community healthcare | industry funding | 5 or less | interviews | R10 |
| Givens [45] | 2006 | Older patients' aversion to antidepressants: A qualitative study | Medical | 3.42 | US | patient | mental health | community healthcare | no pharma involvement | **6** | interviews | R2 |
| Hamrosi [46] | 2006 | Issues with prescribed medications in Aboriginal communities: Aboriginal Health Workers' perspectives | Medical | 0.87 | Developed | clinician | no specific disease | non healthcare | no funding mention | 5 or less | interviews | R10 |
| Hayes [47] | 2006 | Understanding Diabetes Medications From the Perspective of Patients with Type 2 Diabetes | AHP | 1.92 | US | patient | non-communicable disease | combination | industry funding | 6 | focus groups | R3 |
| Morecroft [48] | 2006 | Patients' evaluation of the appropriateness of their hypertension management - A qualitative study | AHP | 2.35 | UK | patient | non-communicable disease | community healthcare | no pharma involvement | 6 | interviews | R7 |
| Veinot [49] | 2006 | "Supposed to make you better but it doesn’t really": HIV-positive youths' perceptions of HIV treatment | AHP | 2.75 | Developed | patient | communicable disease | community healthcare | no pharma involvement | 6 | interviews | R8 |
| Verbeek-Heida [50] | 2006 | Better safe than sorry - why patients prefer to stop selective serotonin reuptake inhibitor (SSRI) antidepressants but are afraid to do so: results of a qualitative study | Social science |  | Developed | patient | mental health | community healthcare | no funding mention | 5 or less | interviews | R2 |
| Angell [51] | 2006 | Promoting treatment adherence in assertive community treatment | Social Science | 2.56 | US | combination | mental health | hospital healthcare | no pharma involvement | 7 | combination | R9 |
| Aronson [52] | 2006 | Antibiotic-taking experiences of undergraduate college students | AHP | 0.87 | US | patient | communicable disease | community healthcare | no pharma involvement | 6 | interviews | R3 |
| Kikkert [53] | 2006 | Medication adherence in schizophrenia: exploring patients', carers' and professionals' views | Medical | 8.61 | Developed | combination | mental health |  | no pharma involvement | 5 or less | focus groups | R2 |
| Seale [54] | 2006 | Sharing decision in consultations involving anti-psychotic mediction: a qualitative study of psychiatrists' experiences | Social science | 2.56 | UK | clinician | mental health | hospital healthcare | no funding mention | 6 | interviews | R7 |
| Bajcar [55] | 2006 | Task analysis of patients' medication-taking practice and the role of making sense: a grounded theory study | AHP | 2.35 | Developed | patient | no specific disease | community healthcare | no funding mention | 6 | interviews | R3 |
| Kremer [56] | 2006 | To tell or not to tell: why people with HIV share or don't share with their physicians whether they are taking their medication as prescribed | Social science | 2.19 | US | patient | communicable disease | community healthcare | industry funding | 6 | interviews | R2 |
| Chen [57] | 2007 | A model of medication taking behaviour in elderly individuals with chronic disease | AHP | 1.81 | Developing | patient | non-communicable disease | hospital healthcare | no pharma involvement | 6 | interviews | R2 |
| Clatworthy [58] | 2007 | Adherence to medication in bipolar disorder: a qualitative study exploring the role of patients' beliefs about the condition and its treatment | Medical | 4.89 | UK | patient | mental health | hospital healthcare | industry funding | 5 or less | interviews | R3 |
| Gordon [59] | 2007 | Effective chronic disease management: Patients' perspectives on medication-related problems | Social science | 2.6 | UK | patient | non-communicable disease | community healthcare | no pharma involvement | 6 | interviews | R6 |
| Interian [60] | 2007 | A qualitative analysis of the perception of stigma among Latinos receiving antidepressants | Medical | 1.99 | US | patient | mental health | community healthcare | industry affiliation/involvement | 6 | focus groups | R4 |
| Campero [61] | 2007 | Bridging the gap between antiretroviral access and adherence in Mexico | Social science | 2.19 | Developing | combination | communicable disease | combination | no pharma involvement | 6 | interviews | R9 |
| Elliott [62] | 2007 | Strategies for coping in a complex world: Adherence behaviour among older adults with chronic disease | Medical | 3.42 | US | patient | no specific disease | community healthcare | no pharma involvement | 6 | interviews | R5 |
| Bane [63] | 2007 | The journey to concordance for patients with hypertension: a qualitative study in primary care | AHP | 1.35 | UK | patient | non-communicable disease | community healthcare | no pharma involvement | 7 | combination | R11 |
| Sidat [64] | 2007 | Experiences and Perceptions with patients with 100% adherence to HAART: a qualitative study | Medical | 3.5 | Developed | patient | communicable disease | hospital healthcare | no funding mention | 6 | interviews | R3 |
| Kagee [65] | 2007 | Treatment adherence among primary care patients in a historically idsadvantaged community in South Africa | Social science | 1.88 | Developing | patient | non-communicable disease | community healthcare | no funding mention | 5 or less | interviews | R8 |
| Piguet [66] | 2007 | Patients' representations of antidepressants: a clue to nonadherence | Medical | 2.7 | Developed | patient | non-communicable disease | hospital healthcare | no funding mention | 6 | interviews | R7 |
| Orr [67] | 2007 | Patient perceptions of factors influencing adherence to medication following kidney transplant | Social science | 1.53 | UK | patient | non-communicable disease | hospital healthcare | no funding mention | 7 | focus groups | R4 |
| Goff [68] | 2008 | Patients' beliefs and preferences regarding doctors' medication recommendations | Medical | 3.42 | US | patient | no specific disease | community healthcare | no pharma involvement | 6 | interviews | R2 |
| Unge [69] | 2008 | Reasons for unsatisfactory acceptance of antiretroviral treatment in the urban Kibera slum, Kenya | Social science | 2.19 | Developing | patient | communicable disease | community healthcare | no pharma involvement | 6 | interviews | R9 |
| Brion [70] | 2008 | Perspectives regarding adherence to prescribed treatment in highly adherent HIV-infected gay men | AHP | 1.23 | US | patient | communicable disease | non healthcare | no pharma involvement | 7 | focus groups | R5 |
| Wrubel [71] | 2008 | Antiretroviral medication support practices among partners of men who have sex with men: a qualitative study | Medical | 3.5 | US | combination | communicable disease | combination | no pharma involvement | 6 | interviews | R1 |
| Fongwa [72] | 2008 | Adherence treatment factors in hypertensive African American women | Medical | 1.59 | US | patient | non-communicable disease | community healthcare | no pharma involvement | 6 | focus groups | R8 |
| Banbury [73] | 2008 | Experiences of analgesic use in patients with low back pain | AHP |  | UK | patient | non-communicable disease | hospital healthcare | no funding mention | 5 or less | interviews | R10 |
| Aspeling [74] | 2008 | Factors associated with adherence to antiretroviral therapy for the treatment of HIV-infection women attending an urban care facility | AHP | 0.54 | Developing | combination | communicable disease |  | no funding mention | 5 or less | interviews | R8 |
| Konkler-Parker [75] | 2008 | Barriers and facilitators to medication adherence in a southern minority population with HIV disease | AHP | 1.23 | US | patient | communicable disease | hospital healthcare | no pharma involvement | 7 | focus groups | R4 |
| Elaine [76] | 2008 | Patients' adherence to osteoporosis therapy. Exploring the perceptions of postmenopausal women | Medical | 1.4 | Developed | patient | non-communicable disease | combination | industry funding | 6 | focus groups | R3 |
| Lehane [77] | 2008 | Medication-taking for coronary artery disease - patients' perspectives | AHP | 1.83 | Developed | patient | non-communicable disease | hospital healthcare | industry funding | 7 | interviews | R3 |
| Miasso [78] | 2008 | Bipolar affective disorder and medication therapy: identifying barriers | AHP | 0.54 | Developing | combination | mental health | hospital healthcare | no funding mention | 5 or less | interviews | R8 |
| Merzel [79] | 2008 | Adherence to antiretroviral therapy among older children and adolescents with HIV: a qualitative study of psychosocial contexts | Medical | 3.5 | US | carer/relative | communicable disease | combination | no pharma involvement | 6 | interviews | R1 |
| Lindberg [80] | 2008 | Overcoming obstacles for adherence to phosphate binding medication in dialysis patients: a qualitative study | AHP | 1.35 | Developed | patient | non-communicable disease | hospital healthcare | no pharma involvement | 6 | interviews | R5 |
| Sanjoobo [81] | 2008 | Barriers and facilitators to patients' adherence to ARV treatment in Zambia: a qualitative study | Social science | 0.81 | Developing | combination | communicable disease | combination | no pharma involvement | 6 | combination | R4 |
| Smith [82] | 2008 | Medicine for chronic illness at school: experience and concerns of young people and their parents | AHP | 1.53 | UK | combination | non-communicable disease | hospital healthcare | no pharma involvement | 6 | interviews | R4 |
| Sabin [83] | 2008 | Barriers to adherence to antiretroviral medications among patients living with HIV in Southern China: a qualitative study | Social science | 2.19 | Developing | patient | communicable disease | hospital healthcare | no pharma involvement | 6 | combination | R9 |
| Bokhour [84] | 2008 | Patterns of concordance and non-concordance with clinicians recommendations and parents' explanatory models | Social science | 2.6 | US | carer/relative | non-communicable disease | combination | no pharma involvement | 6 | interviews | R3 |
| Beusterien [85] | 2008 | HIV patients insight on adhering to medication: a qualitative analysis | Social science | 2.19 | US | patient | communicable disease | combination | industry funding | 7 | focus groups | R4 |
| dosReis [86] | 2009 | The meaning of Attention-Deficit Hyperactivity Disorder medication and parents initiation and continuity | Medical | 3.07 | US | carer/relative | mental health | hospital healthcare | no pharma involvement | 6 | interviews | R1 |
| Chong [87] | 2009 | What affects asthma medicine use in children? Australian asthma educator perspectives | Medical | 1.83 | Developed | clinician | non-communicable disease | combination | no pharma involvement | 6 | combination | R8 |
| Garavalia [88] | 2009 | Exploring patients' reasons for discontinuance of heart medications | AHP | 1.81 | US | patient | non-communicable disease | hospital healthcare | industry funding | 6 | interviews | R2 |
| Granger [89] | 2009 | A qualitative descriptive study of the work of adherence to a chronic heart failure regimen | AHP | 1.81 | US | combination | non-communicable disease | hospital healthcare | no pharma involvement | 6 | interviews | R4 |
| Gusdal [90] | 2009 | Voices on adherence to ART in Ethiopia and Uganda: a matter of choice or simply not an option? | Social science | 2.19 | Developed | combination | communicable disease | community healthcare | no pharma involvement | 6 | interviews | R9 |
| Brinkman [91] | 2009 | Parental angst making and revisiting decisions about treatment of attention-deficit/hyperactivity disorder | Medical | 5.29 | US | carer/relative | mental health | community healthcare | industry funding | 6 | focus groups | R5 |
| Hansen [92] | 2009 | "I'd rather not take it, but… ": Young women's perceptions of medicines | Social science | 2.19 | Developed | patient | no specific disease | non healthcare | no pharma involvement | 7 | interviews | R4 |
| Haslbeck [93] | 2009 | Routines in medication management: the perspective of people with chronic conditions | Social science |  | Developed | patient | non-communicable disease | combination | no pharma involvement | 6 | interviews | R5 |
| Hughes [94] | 2009 | "I just take what I am given" Adherence and resident involvement in decision making on medicines in nursing homes for older people | Medical | 2.5 | UK | combination | no specific disease | community healthcare | no pharma involvement | 7 | combination | R7 |
| Vervoort [95] | 2009 | Adherence to HAART: processes explaining adherence behaviour in acceptors and non-acceptors | Social science | 2.19 | Developed | patient | communicable disease | hospital healthcare | no funding mention | 7 | interviews | R3 |
| Vreeman [96] | 2009 | Factors sustaining paediatric adherence to antiretroviral therapy in western Kenya | Social science | 2.19 | Developing | carer/relative | communicable disease | community healthcare | no pharma involvement | 6 | combination | R9 |
| Watt [97] | 2009 | "It's all the time in my mind": Facilitators of adherence to antiretroviral therapy in a Tanzanian setting | Social science | 2.56 | Developing | combination | communicable disease | hospital healthcare | no pharma involvement | 5 or less | interviews | R1 |
| Williams [98] | 2009 | The role of irrational thought in medicine adherence: people with diabetic kidney disease | AHP | 1.69 | Developed | patient | non-communicable disease | hospital healthcare | no pharma involvement | 6 | interviews | R5 |
| Kelly [99] | 2009 | Patients with dysphagia: experiences of taking medication | AHP | 1.69 | UK | patient | non-communicable disease | community healthcare | no pharma involvement | 6 | interviews | R4 |
| Kourrouski [100] | 2009 | Treatment adherence: the experience of adolescents with HIV/AIDs | AHP | 0.54 | Developing | patient | communicable disease | hospital healthcare | no funding mention | 6 | interviews | R9 |
| Lacey [101] | 2009 | Barriers to adherence with glaucoma medications: a qualitative research study | Medical | 1.9 | UK | patient | non-communicable disease | hospital healthcare | no pharma involvement | 7 | combination | R3 |
| McMullen [102] | 2009 | Women's accounts of their decision to quit taking antidepressants | Social science | 2.19 | Developed | patient | mental health | non healthcare | no pharma involvement | 7 | interviews | R2 |
| Stewart [103] | 2009 | It is not my job, I'm the patient not the doctor': patient perspectives on medicines management in the treatment of schizophrenia | Social science | 2.6 | UK | patient | mental health | hospital healthcare | no pharma involvement | 6 | interviews | R3 |
| Ruppar [104] | 2009 | Medication adherence in successful kidney transplant recipients | Medical | 0.69 | US | patient | non-communicable disease | hospital healthcare | no pharma involvement | 6 | interviews | R4 |
| Murray [105] | 2009 | Barriers to acceptance and adherence of antiretroviral therapy in urban Zambian women: a qualitative study | Social science | 2.19 | Developing | combination | communicable disease |  | no pharma involvement | 5 or less | interviews | R4 |
| Biadgilign [106] | 2009 | Barriers and facilitators to antiretroviral mediation adherence among HIV-infected paediatric patients in Ethiopia: a qualitative study | Social science | 0.81 | Developing | combination | communicable disease | hospital healthcare | no pharma involvement | 6 | interviews | R4 |
| Stevens [107] | 2009 | Pill taking from the perspective of HIV-infected women who are vulnerable to anti-retroviral treatment failure | Social science | 2.19 | US | patient | communicable disease | community healthcare | no pharma involvement | 6 | interviews | R7 |
| Curioso [108] | 2010 | Understanding the facilitators and barriers of antiretroviral adherence in Peru | Medical | 2.32 | Developing | patient | non-communicable disease | community healthcare | no pharma involvement | 6 | interviews | R4 |
| Guimaraes [109] | 2010 | Exploring patients' perceptions for insulin therapy in type 2 diabetes: a Brazilian and Canadian qualitative study | Social science | 2.6 | Developing | patient | non-communicable disease | combination | no pharma involvement | 6 | combination | R5 |
| Harrold [110] | 2010 | Patients and providers view gout differently: a qualitative study | Social science |  | US | combination | non-communicable disease | community healthcare | no pharma involvement | 6 | interviews | R5 |
| Hill-Smith [111] | 2010 | Involving patients in decisions about preventive medication: a focus group study | Medical |  | UK | patient | non-communicable disease | community healthcare | no pharma involvement | 6 | focus groups | R3 |
| Howes [112] | 2010 | Barriers to diagnosing and managing hypertension: A qualitative study in Australian general practice | Medical | 0.67 | Developed | clinician | non-communicable disease | community healthcare | no pharma involvement | 7 | focus groups | R10 |
| Lewis [113] | 2010 | Medication adherence beliefs of community-dwelling hypertensive African Americans | AHP | 1.81 | US | patient | non-communicable disease | community healthcare | no pharma involvement | 7 | focus groups | R4 |
| Michaud [114] | 2010 | Coping with an HIV infection | Medical | 1.88 | Developed | patient | communicable disease | hospital healthcare | no pharma involvement | 7 | interviews | R2 |
| Mohammedpour [115] | 2010 | HIV-infected patients' adherence to highly active antiretroviral therapy: A phenomenological study | AHP | 0.85 | Developing | patient | communicable disease | hospital healthcare | no pharma involvement | 6 | interviews | R7 |
| Wang [116] | 2010 | Bipolar disorder and medical adherence: A Chinese perspective | Medical |  | Developed | patient | mental health | community healthcare | no pharma involvement | 6 | interviews | R2 |
| Duxbury [117] | 2010 | Administration of medication in the acute mental health ward | AHP | 2 | UK | combination | mental health | hospital healthcare | no pharma involvement | 6 | interviews | R10 |
| Wai [118] | 2010 | Perspectives on adherence to blood pressure - lowering medications among Samoan patients: qualitative interviews | Medical |  | Developed | patient | non-communicable disease | community healthcare | no pharma involvement |  | interviews | R10 |
| Abrahams [119] | 2010 | Barriers to post exposure prophylaxis completion after rape: a South African qualitative study | Social science | 1.55 | Developing | patient | communicable disease | community healthcare | no funding mention | 6 | interviews | R4 |
| Bolster [120] | 2010 | Person-centred interactions between nurses and patients during medication activities in an acute hospital setting | AHP | 2.25 | Developed | combination | no specific disease | hospital healthcare | no pharma involvement | 7 | combination | R7 |
| Rifkin [121] | 2010 | Medication adherence behaviour and priorities among older adults with CKD: a semi structured interview study | Medical | 5.76 | US | patient | non-communicable disease | hospital healthcare | no pharma involvement | 6 | interviews | R2 |
| Matlock [122] | 2010 | Patient perspectives on decision making in heart failure | Medical | 3.07 | US | patient | non-communicable disease | hospital healthcare | no pharma involvement | 5 or less | interviews | R2 |
| Chambers [123] | 2011 | Adherence to medication in stroke survivors: A qualitative comparison of low and high adherers | AHP | 2.7 | UK | patient | non-communicable disease | hospital healthcare | no pharma involvement | 6 | interviews | R4 |
| Fredriksen-Goldren [124] | 2011 | "You must take the medications for you and for me": Family caregivers promoting HIV medication adherence in China | Medical | 3.5 | US | combination | communicable disease | hospital healthcare | no pharma involvement | 7 | interviews | R1 |
| Garavalia [125] | 2011 | Clinician-patient discord: explaining differences in perspectives for discontinuing clopidogrel | AHP | 1.83 | US | combination | non-communicable disease | hospital healthcare | industry funding | 6 | interviews | R8 |
| Grant [126] | 2011 | Diabetes oral medication initiation and intensification: Patient views compared with current treatment guidelines | AHP | 1.92 | US | patient | non-communicable disease | community healthcare | no pharma involvement | 6 | focus groups | R2 |
| Hommel [127] | 2011 | Treatment adherence in paediatric inflammatory bowel disease: perceptions from adolescent patients and their families | Social science | 0.86 | US | combination | non-communicable disease | hospital healthcare | no pharma involvement | 6 | interviews | R4 |
| Iversen [128] | 2011 | Factors affecting adherence to osteoporosis medications: A focus group approach examining viewpoints of patients and providers | AHP | 1.26 | US | combination | non-communicable disease | hospital healthcare | no pharma involvement | 5 or less | interviews | R10 |
| Milder [129] | 2011 | "It looks after me": How older patients make decisions about analgesics for osteoarthritis | Medical | 4.04 | Developed | patient | non-communicable disease | community healthcare | industry affiliation/involvement | 6 | interviews | R7 |
| Mills [130] | 2011 | Prisoners' experiences of antipsychotic medication: influences on adherence | Medical | 0.88 | UK | patient | mental health | non healthcare | no pharma involvement | 6 | interviews | R3 |
| Toverud [131] | 2011 | Norwegian patients on generic antihypertensive drugs: a qualitative study of their own experiences | Medical | 2.7 | Developed | patient | non-communicable disease | community healthcare | no funding mention | 5 or less | focus groups | R2 |
| Tranulis [132] | 2011 | Becoming adherent to antipsychotics: A qualitative study of treatment-experienced schizophrenia patients | Medical | 1.99 | US | patient | mental health | community healthcare | industry funding | 6 | interviews | R3 |
| van Geffen [133] | 2011 | The decision to continue or discontinue treatment: Experiences and beliefs of users of selective serotonin-reuptake inhibitors in the initial months-A qualitative study | AHP | 2.35 | Developed | patient | mental health | community healthcare | no pharma involvement | 6 | interviews | R4 |
| Van Tam [134] | 2011 | "It is not that I forget, it's just that I don’t want other people to know": barriers to and strategies for adherence to atiretroviral therapy among HIV patients in Northern Vietnam | Social science | 2.19 | Developed | combination | communicable disease | hospital healthcare | no pharma involvement | 6 | focus groups | R6 |
| Wamboldt [135] | 2011 | Adolescent decision-making about use of inhaled asthma controller medication: Results from focus groups with participants from a prior longitudinal study | Medical | 1.83 | US | patient | non-communicable disease | non healthcare | no pharma involvement | 7 | focus groups | R7 |
| Watermeyer [136] | 2011 | "Now here comes the pills that are going to save your life": pharmacists' discussions of antiretroviral drugs in a context of life and death | Social science | 2.19 | Developing | combination | communicable disease | community healthcare | no pharma involvement | 6 | observation | R11 |
| Williams [137] | 2011 | Qualitative assessment of barriers and facilitators to HIV treatment | AHP | 1.23 | US | patient | communicable disease | hospital healthcare | no pharma involvement | 7 | focus groups | R5 |
| Ujiji [138] | 2011 | Reasoning and deciding PMTCT adherence during pregnancy among women living with HIV in Kenya | Social science | 1.55 | Developing | patient | communicable disease | combination | no pharma involvement | 6 | interviews | R9 |
| Axelsson [139] | 2011 | Motivational foci and asthma medication tactics directed toward a functional day | Medical | 2.32 | Developed | patient | non-communicable disease |  | no pharma involvement | 7 | interviews | R3 |
| Kranke [140] | 2011 | A qualitative investigation of self-stigma among adolescents taking psychiatric medication | Medical | 1.99 | US | patient | mental health | combination | no pharma involvement | 6 | interviews | R3 |
| Klok [141] | 2011 | Parental illness perceptions and medication perceptions in childhood asthma, a focus group study | Medical | 1.84 | Developed | carer/relative | non-communicable disease | combination | no pharma involvement | 6 | focus groups | R7 |
| Sale [142] | 2011 | Decision to take osteoporosis medication in patients who have had a fracture and are 'high' risk for future fracture: a qualitative study | Medical | 1.9 | Developed | patient | non-communicable disease | hospital healthcare | no pharma involvement | 6 | interviews | R5 |
| Salt [143] | 2011 | The complexity of the treatment: the decision-making process among women with RA | Social science | 2.19 | US | patient | non-communicable disease | hospital healthcare | no pharma involvement | 6 | interviews | R8 |
| Stumbo [144] | 2011 | A qualitative study of HIV treatment adherence support from friends and family among same sex male couples | AHP |  | US | combination | communicable disease | combination | no pharma involvement | 5 or less | interviews | R1 |
| Penn [145] | 2011 | Why don’t patients take their drugs? The role of communication, context and culture in patient adherence and the work of the pharmacist in HIV/AIDS | Social science | 2.6 | Developing | combination | communicable disease | combination | no pharma involvement | 5 or less | combination | R7 |
| Arrivillaga [146] | 2011 | Applying and expanded social determinant approach to the concept of adherence to treatment: the case of Columbian women living with HIV/AIDS | Medical | 2.33 | Developing | patient | communicable disease | community healthcare | no pharma involvement | 6 | combination | R9 |
| Badahdah [147] | 2011 | "I want to stand on my own legs" a qualitative study of ART adherence among HIV-positive women in Egypt | Social science | 2.19 | Developing | patient | communicable disease |  | no pharma involvement | 6 | interviews | R7 |
| Ballantyne [148] | 2011 | Becoming old as a 'pharmaceutical person': negotiation of health and medicines among ethnocululturally diverse older adults | Medical | 0.92 | Developed | patient | no specific disease | non healthcare | no pharma involvement | 7 | interviews | R2 |
| Rust [149] | 2011 | Health Literacy and medication adherence in undeserved African-American breast cancer survivors: a qualitative study | Social science | 0.62 | US | patient | non-communicable disease | non healthcare | no funding mention | 5 or less | focus groups | R10 |
| Armitage [150] | 2011 | Exploring the delivery of antiretroviral therapy for symptomatic HIV in Swaziland: threats to the successful treatment and safety of outpatients attending regional and district clinics | Medical | 3.28 | Developing | combination | communicable disease | hospital healthcare | no funding mention | 7 | combination | R9 |
| Landier [151] | 2011 | A grounded theory of the process of adherence to oral chemotherapy in Hispanic and Caucasian children and adolescents | AHP | 0.87 | US | combination | non-communicable disease | hospital healthcare | no pharma involvement | 7 | combination | R6 |
| Boorgsteede [152] | 2011 | Factors related to high and low levels of adherence according to patients with type 2 diabetes | AHP | 1.27 | Developed | patient | non-communicable disease | community healthcare | no pharma involvement | 6 | interviews | R4 |
| Decker [153] | 2012 | Exploring barriers to optimal anticoagulation for atrial fibrillation | AHP | 0 | US | clinician | non-communicable disease | hospital healthcare | industry funding | 6 | interviews | R11 |
| Colleti [154] | 2012 | Parent perspectives on the decision to initiate medication treatment of Attention-Deficit/Hyperactivity Disorder | Medical | 3.07 | US | carer/relative | mental health | hospital healthcare | industry affiliation/involvement | 7 | focus groups | R4 |
| Cormier [155] | 2012 | How Parents make decisions to use medications to treat their child's ADHD: A grounded theory study | AHP |  | US | carer/relative | mental health | non healthcare | no pharma involvement | 6 | interviews | R1 |
| Granger [156] | 2012 | The meanings associated with medicines in heart failure patients | AHP | 1.83 | US | patient | non-communicable disease | hospital healthcare | no pharma involvement | 6 | interviews | R6 |
| Hon [157] | 2012 | Factors influencing the adherence of antipsychotic medication (Aripiprazole) in first-episode psychosis: findings from a grounded theory study | AHP | 0.84 | UK | patient | mental health | hospital healthcare | no funding mention | 6 | interviews | R4 |
| Brinkman [158] | 2012 | In their own words: adolescent views on ADHD and their evolving role managing medication | Medical | 2 | US | patient | mental health | community healthcare | industry funding | 7 | focus groups | R6 |
| Jaarsma [159] | 2012 | Nurses' strategies to address self-care aspects related to medication adherence and symptom recognition in heart failure patients: An in-depth look | Medical | 1.32 | Developed | clinician | non-communicable disease | combination | industry funding | 5 or less | combination | R8 |
| van den Boogard [160] | 2012 | An exploration of patient perceptions of adherence to tuberculosis treatment in Tanzania | Social science | 2.19 | Developing | patient | communicable disease | community healthcare | no pharma involvement | 7 | interviews | R8 |
| Vilhelmsson [161] | 2012 | Experiences from consumer reports on psychiatric adverse drug reactions with antidepressant medication: a qualitative study of reports to a consumer association | Medical | 1.84 | Developed | patient | mental health | non healthcare | no pharma involvement | 6 | combination | R8 |
| Buus [162] | 2012 | Explanatory models of depression and treatment adherence to antidepressant medication: A qualitative interview study | AHP | 2.25 | Developed | patient | mental health | hospital healthcare | no pharma involvement | 7 | interviews | R6 |
| Wasti [163] | 2012 | Barriers to and facilitators of antiretroviral therapy adherence in Nepal: A qualitative study | Medical | 1.39 | Developing | combination | communicable disease | community healthcare | no pharma involvement | 6 | interviews | R4 |
| Watermeyer [164] | 2012 | "Only two months destroys everything": A case study of communication about nonadherence to antiretroviral therapy in a South African HIV pharmacy context | Social science | 1.61 | Developing | combination | communicable disease | community healthcare | no funding mention | 6 | combination | R11 |
| Watermeyer [165] | 2012 | "This clinic is number one": A qualitative study of factors that contribute toward "successful" care at a South African pediatric HIV/AIDS Clinic | AHP | 1.67 | Developing | combination | communicable disease | combination | no pharma involvement | 5 or less | interviews | R7 |
| Wendorf [166] | 2012 | Navigating hazardous conditions: Understanding HIV medication adherence in the context of depression | Social science | 2.19 | US | patient | communicable disease | hospital healthcare | no pharma involvement | 7 | interviews | R4 |
| Widnes [167] | 2012 | Risk perception and medicines information needs in pregnant women with epilepsy - A qualitative study | Medical | 2.06 | Developed | patient | non-communicable disease | hospital healthcare | no funding mention | 7 | interviews | R6 |
| Drey [168] | 2012 | Adherence to antiparkinsonian medication: An in depth qualitative study | AHP | 2.24 | UK | patient | non-communicable disease | hospital healthcare | no pharma involvement | 7 | interviews | R3 |
| Arrivillaga [169] | 2012 | HIV/AIDS treatment adherence in economically better off women in Columbia | Social science | 2.19 | Developing | patient | communicable disease | community healthcare | no pharma involvement | 7 | focus groups | R9 |
| Besser [170] | 2012 | How do osteoporosis patients perceive their illness and treatment? | Medical | 4.17 | UK | patient | non-communicable disease | hospital healthcare | no pharma involvement | 6 | combination | R8 |
| Rushworth [171] | 2012 | Patient-specific factors relating to medication adherence in a post-percutaneous coronary intervention cohort | AHP |  | UK | patient | non-communicable disease | hospital healthcare | no pharma involvement | 7 | interviews | R3 |
| Kucukarslan [172] | 2012 | Exploring patient experiences with prescription medicines to identify unmet patient needs: implications for research and practice | AHP | 2.35 | US | patient | non-communicable disease | community healthcare | no pharma involvement | 5 or less | focus groups | R6 |
| Lemp [173] | 2012 | Patients' views about treatment with combination therapy for RA: a comparative qualitative study | Medical | 1.9 | UK | patient | non-communicable disease | hospital healthcare | no funding mention | 6 | interviews | R3 |
| Matovu [174] | 2012 | Narratives of Ugandan Women adhering to HIV/AIDS medication | AHP | 0.67 | Developing | patient | communicable disease |  | no pharma involvement | 5 or less | interviews | R3 |
| Rogers [175] | 2012 | "I think we're all guinea pigs really": a qualitative study of medication and borderline personality disorder | AHP | 0.98 | UK | patient | mental health | hospital healthcare | no funding mention | 6 | interviews | R10 |
| Brown [176] | 2012 | Development of a conceptual model of adherence to oral anticoagulants to reduce risk of stroke in patients with atrial fibrillation | AHP | 2.68 | US | patient | non-communicable disease |  | industry funding | 6 | focus groups | R8 |
| Bezreh [177] | 2012 | Challenges to physician-patient communication about medication use: a window in the sceptical world | Social science | 2.6 | US | patient | no specific disease | non healthcare | no pharma involvement | 7 |  | R2 |
| Bassett-Clarke [178] | 2012 | Ethnic differences in medicines-taking in older adults: a cross-cultural study in New Zealand | AHP |  | Developed | patient | no specific disease | combination | no pharma involvement | 6 | focus groups | R3 |
| Lee [179] | 2012 | A qualitative study on health care professionals' perceived barriers to insulin initiations in a multi-ethnic population | Medical | 1.74 | Developing | clinician | non-communicable disease | combination | no pharma involvement | 6 | combination | R8 |
| Read [180] | 2012 | "I want the one that will heal me completely so it won't come back again": the limits of antipsychotic medication in rural Ghana | Medical | 1.52 | Developing | combination | mental health | combination | no pharma involvement | 5 or less | combination | R9 |
| Santer [181] | 2012 | Experiences of carers managing childhood eczema and their views on its treatment: a qualitative study | Medical | 2.36 | UK | carer/relative | non-communicable disease | community healthcare | no pharma involvement | 5 or less | interviews | R5 |
| Chang [182] | 2013 | Qualitative inquiry into motivators for maintaining medication adherence among Taiwanese with schizophrenia | AHP | 2.01 | Developing | patient | mental health | combination | no pharma involvement | 7 | interviews | R3 |
| Flynn [183] | 2013 | Facilitators and barriers to hypertension self management | Social science | 2.6 | US | combination | non-communicable disease | community healthcare | no pharma involvement | 7 | focus groups | R6 |
| Griva [184] | 2013 | Managing treatment for end-stage renal disease - A qualitative study exploring cultural perspectives on facilitators and barriers to treatment adherence | Social science | 1.95 | Developed | patient | non-communicable disease | hospital healthcare | no pharma involvement | 6 | combination | R3 |
| Grover [185] | 2013 | Medication use in Indian children with asthma: The user's perspective | Medical | 3.5 | Developed | combination | non-communicable disease | hospital healthcare | no funding mention | 5 or less | interviews | R9 |
| Brion [186] | 2013 | Grief and HIV medication adherence: the work of transcending loss | Social science | 1.03 | US | patient | communicable disease | community healthcare | no funding mention | 6 | focus groups | R2 |
| Walstrom [187] | 2013 | I think my future will be better than my past': Examining support group influence on the mental health of HIV-infected Rwandan women | Medical | 0.92 | US | patient | communicable disease | hospital healthcare | no pharma involvement | 7 | focus groups | R6 |
| Waterman [188] | 2013 | Adherence to ocular hypotensive therapy: patient health education needs and views on group education | Social science | 2.6 | UK | patient | non-communicable disease | hospital healthcare | no funding mention | 7 | interviews | R8 |
| Stamer [189] | 2013 | Non-compliance: a never-ending story. Understanding the perspective of patients with rheumatoid arthritis | Social science |  | Developed | patient | non-communicable disease | hospital healthcare | industry funding | 6 | interviews | R7 |
| Teferra [190] | 2013 | Perspectives on reasons for non-adherence to medication in persons with schizophrenia in Ethiopia | Medical | 2.24 | Developing | combination | mental health | hospital healthcare | no pharma involvement | 7 | combination | R9 |
| Murdoch [191] | 2013 | Resisting medications: moral discourses and performances in illness narratives | Social science | 1.88 | UK | patient | non-communicable disease |  | no pharma involvement | 7 | interviews | R3 |
| Stewart [192] | 2013 | Medication-taking beliefs and diabetes in American Samoa: a qualitative inquiry | Social science |  | US | combination | non-communicable disease | community healthcare | no pharma involvement | 6 | combination | R4 |

1. Morgan M, Watkins CJ. Managing hypertension: beliefs and responses to medication among cultural groups. Sociology of Health & Illness. 1988 Dec 1;10(4):561-78.

2. Karp DA. Taking anti-depressant medications: Resistance, trial commitment, conversion, disenchantment. Qualitative Sociology. 1993 Dec 1;16(4):337-59.

3. Dowell JO, Hudson H. A qualitative study of medication-taking behaviour in primary care. Family Practice. 1997 Oct 1;14(5):369-75.

4. Adams S, Pill R, Jones A. Medication, chronic illness and identity: the perspective of people with asthma. Social science & medicine. 1997 Jul 1;45(2):189-201

5. Boath EH, Blenkinsopp A. The rise and rise of proton pump inhibitor drugs: patients' perspectives. Social Science & Medicine. 1997 Nov 1;45(10):1571-9.

6. Lisper L, Isacson D, Sjödén PO, Bingefors K. Medicated hypertensive patients' views and experience of information and communication concerning antihypertensive drugs. Patient education and counseling. 1997 Nov 1;32(3):147-55.

7. Johnson MJ, Williams M, Marshall ES. Adherent and nonadherent medication-taking in elderly hypertensive patients. Clinical nursing research. 1999 Nov;8(4):318-35.

8. Chen J, Britten N. ‘Strong medicine’: an analysis of pharmacist consultations in primary care. Family practice. 2000 Dec 1;17(6):480-3.

9. Boyle E, Chambers M. Medication compliance in older individuals with depression: gaining the views of family carers. Journal of psychiatric and mental health nursing. 2000 Dec 1;7(6):515-22.

10. Francis SA, Patel M. Caring for people with schizophrenia: family carers' involvement with medication. International Journal of Pharmacy Practice. 2000 Dec 1;8(4):314-24.

11. Ivers H, McGrath PJ, Purdy RA, Hennigar AW, Campbell MA. Decision making in migraine patients taking sumatriptan: an exploratory study. Headache: The Journal of Head and Face Pain. 2000 Feb 1;40(2):129-36.

12. Svensson S, Kjellgren KI, Ahlner J, Säljö R. Reasons for adherence with antihypertensive medication. International journal of cardiology. 2000 Dec 31;76(2):157-63.

13. Usher K. Taking neuroleptic medications as the treatment for schizophrenia: A phenomenological study. Australian and New Zealand Journal of Mental Health Nursing. 2001 Sep 1;10(3):145-55.

14. Angermeyer MC, Löffler W, Müller P, Schulze B, Priebe S. Patients' and relatives' assessment of clozapine treatment. Psychological medicine. 2001 Apr 1;31(03):509-17.

15. Golin C, Isasi F, Bontempi JB, Eng E. Secret pills: HIV-positive patients' experiences taking antiretroviral therapy in North Carolina. AIDS Education and Prevention. 2002 Aug 1;14(4):318-29.

16. Happell B, Manias E, Pinikahana J. The role of the inpatient mental health nurse in facilitating patient adherence to medication regimes. International Journal of Mental Health Nursing. 2002 Dec 1;11(4):251-9.

17. Wilson HS, Hutchinson SA, Holzemer WL. Reconciling incompatibilities: a grounded theory of HIV medication adherence and symptom management. Qualitative Health Research. 2002 Dec 1;12(10):1309-22.

18. Benson J, Britten N. Patients' decisions about whether or not to take antihypertensive drugs: qualitative study. Bmj. 2002 Oct 19;325(7369):873.

19. Knudsen P, Hansen EH, Traulsen JM. Perceptions of young women using SSRI antidepressants—a reclassification of stigma. International journal of pharmacy practice. 2002 Dec 1;10(4):243-52.

20. Jones SG. The other side of the pill bottle: the lived experience of HIV-positive nurses on HIV combination drug therapy. Journal of the Association of Nurses in AIDS Care. 2002 Jun 30;13(3):22-36.

21. Sankar A, Luborsky M, Schuman P, Roberts G. Adherence discourse among African-American women taking HAART. AIDS care. 2002 Apr 1;14(2):203-18.

22. Kärner A, Göransson A, Bergdahl B. Conceptions on treatment and lifestyle in patients with coronary heart disease—a phenomenographic analysis. Patient education and counseling. 2002 Jun 30;47(2):137-43.

23. Garfield S, Smith F, Francis SA. The paradoxical role of antidepressant medication-returning to normal functioning while losing the sense of being normal. Journal of Mental Health. 2003 Jan 1;12(5):521-35.

24. Brackis-Cott E, Mellins CA, Abrams E, Reval T, Dolezal C. Pediatric HIV medication adherence: the views of medical providers from two primary care programs. Journal of Pediatric Health Care. 2003 Oct 31;17(5):252-60.

25. Russell CL, Kilburn E, Conn VS, Libbus MK, Ashbaugh C. Medication-taking beliefs of adult renal transplant recipients. Clinical Nurse Specialist. 2003 Jul 1;17(4):200-8.

26. Unson CG, Siccion E, Gaztambide J, Gaztambide S, Mahoney Trella P, Prestwood K. Nonadherence and osteoporosis treatment preferences of older women: a qualitative study. Journal of Women's Health. 2003 Dec 1;12(10):1037-45.

27. Lukoschek P. African Americans' beliefs and attitudes regarding hypertension and its treatment: a qualitative study. Journal of Health Care for the Poor and Underserved. 2003;14(4):566-87.

28. Remien RH, Hirky AE, Johnson MO, Weinhardt LS, Whittier D, Le GM. Adherence to medication treatment: A qualitative study of facilitators and barriers among a diverse sample of HIV+ men and women in four US cities. AIDS and Behavior. 2003 Mar 1;7(1):61-72.

29. Knudsen P, Hansen EH, Eskildsen K. Leading ordinary lives: a qualitative study of younger women's perceived functions of antidepressants. Pharmacy World and Science. 2003 Aug 1;25(4):162-7.

30. Carrick R, Mitchell A, Powell RA, Lloyd K. The quest for well‐being: A qualitative study of the experience of taking antipsychotic medication. Psychology and Psychotherapy: Theory, Research and Practice. 2004 Mar 1;77(1):19-33.

31. Gascón JJ, Sánchez-Ortuño M, Llor B, Skidmore D, Saturno PJ, Treatment Compliance in Hypertension Study Group. Why hypertensive patients do not comply with the treatment Results from a qualitative study. Family practice. 2004 Apr 1;21(2):125-30.

32. Happell B, Manias E, Roper C. Wanting to be heard: mental health consumers’ experiences of information about medication. International journal of mental health nursing. 2004 Dec 1;13(4):242-8.

33. Haslam C, Brown S, Atkinson S, Haslam R. Patients' experiences of medication for anxiety and depression: effects on working life. Family Practice. 2004 Apr 1;21(2):204-12.

34. Wood SA, Tobias C, McCree J. Medication adherence for HIV positive women caring for children: in their own words. AIDS care. 2004 Oct 1;16(7):909-13.

35. Penza‐Clyve SM, Mansell C, McQuaid EL. Why don't children take their asthma medications? A qualitative analysis of children's perspectives on adherence. Journal of Asthma. 2004 Jan 1;41(2):189-97.

36. Bollini P, Tibaldi G, Testa C, Munizza C. Understanding treatment adherence in affective disorders: a qualitative study. Journal of psychiatric and mental health nursing. 2004 Dec 1;11(6):668-74.

37. Deegan PE. The importance of personal medicine: A qualitative study of resilience in people with psychiatric disabilities. Scandinavian Journal of Public Health. 2005 Oct 1;33(66 suppl):29-35.

38. Connell P, McKevitt C, Wolfe C. Strategies to manage hypertension: a qualitative study with black Caribbean patients. Br J Gen Pract. 2005 May 1;55(514):357-61.

39. Halkitis PN, Shrem MT, Zade DD, Wilton L. The physical, emotional and interpersonal impact of HAART: exploring the realities of HIV seropositive individuals on combination therapy. Journal of health psychology. 2005 May;10(3):345-58.

40. Wrubel J, Moskowitz JT, Richards TA, Prakke H, Acree M, Folkman S. Pediatric adherence: perspectives of mothers of children with HIV. Social science & medicine. 2005 Dec 31;61(11):2423-33.

41. Albus C, Schmeißer N, Salzberger B, Fätkenheuer G. Preferences regarding medical and psychosocial support in HIV-infected patients. Patient education and counseling. 2005 Jan 31;56(1):16-20.

42. Morgan M, Figueroa-Muñoz JI. Barriers to uptake and adherence with malaria prophylaxis by the African community in London, England: focus group study. Ethnicity and Health. 2005 Nov 1;10(4):355-72.

43. Kumarasamy N, Safren SA, Raminani SR, Pickard R, James R, Krishnan AS, Solomon S, Mayer KH. Barriers and facilitators to antiretroviral medication adherence among patients with HIV in Chennai, India: a qualitative study. AIDS Patient Care & STDs. 2005 Aug 1;19(8):526-37.

44. Badger F, Nolan P. Concordance with antidepressant medication in primary care. Nursing standard. 2006 Sep 6;20(52):35-40.

45. Givens JL, Datto CJ, Ruckdeschel K, Knott K, Zubritsky C, Oslin DW, Nyshadham S, Vanguri P, Barg FK. Older patients' aversion to antidepressants. Journal of General Internal Medicine. 2006 Feb 1;21(2):146-51.

46. Hamrosi K, Taylor SJ, Aslani P. Issues with prescribed medications in Aboriginal communities: Aboriginal health workers’ perspectives. Rural Remote Health. 2006 May 9;6(2):557.

47. Hayes RP, Bowman L, Monahan PO, Marrero DG, McHorney CA. Understanding Diabetes Medications From the Perspective of Patients With Type 2 Diabetes Prerequisite to Medication Concordance. The Diabetes Educator. 2006 May 1;32(3):404-14.

48. Morecroft C, Cantrill J, Tully MP. Patients' evaluation of the appropriateness of their hypertension management—A qualitative study. Research in Social and Administrative Pharmacy. 2006 Jun 30;2(2):186-211.

49. Veinot TC, Flicker SE, Skinner HA, McClelland A, Saulnier P, Read SE, Goldberg E. “Supposed to make you better but it doesn’t really”: HIV-positive youths’ perceptions of HIV treatment. Journal of Adolescent Health. 2006 Mar 31;38(3):261-7.

50. Verbeek-Heida PM, Mathot EF. Better safe than sorry—why patients prefer to stop using selective serotonin reuptake inhibitor (SSRI) antidepressants but are afraid to do so: results of a qualitative study. Chronic Illness. 2006 Jun;2(2):133-42.

51. Angell B, Mahoney CA, Martinez NI. Promoting treatment adherence in assertive community treatment. Social Service Review. 2006 Sep;80(3):485-526.

52. Aronson BS. Antibiotic‐taking experiences of undergraduate college students. Journal of the American Association of Nurse Practitioners. 2006 Dec 1;18(12):591-8.

53. Kikkert MJ, Schene AH, Koeter MW, Robson D, Born A, Helm H, Nose M, Goss C, Thornicroft G, Gray RJ. Medication adherence in schizophrenia: exploring patients', carers' and professionals' views. Schizophrenia Bulletin. 2006 Oct 1;32(4):786-94.

54. Seale C, Chaplin R, Lelliott P, Quirk A. Sharing decisions in consultations involving anti-psychotic medication: a qualitative study of psychiatrists’ experiences. Social science & medicine. 2006 Jun 30;62(11):2861-73.

55. Bajcar J. Task analysis of patients' medication-taking practice and the role of making sense: a grounded theory study. Research in Social and Administrative Pharmacy. 2006 Mar 31;2(1):59-82.

56. Kremer H, Ironson G. To tell or not to tell: Why people with HIV share or don't share with their physicians whether they are taking their medications as prescribed. AIDS care. 2006 Jul 1;18(5):520-8.

57. Chen CH, Wu JR, Yen M, Chen ZC. A model of medication-taking behavior in elderly individuals with chronic disease. Journal of Cardiovascular Nursing. 2007 Sep 1;22(5):359-65.

58. Clatworthy J, Bowskill R, Rank T, Parham R, Horne R. Adherence to medication in bipolar disorder: a qualitative study exploring the role of patients’ beliefs about the condition and its treatment. Bipolar disorders. 2007 Sep 1;9(6):656-64.

59. Gordon K, Smith F, Dhillon S. Effective chronic disease management: patients’ perspectives on medication-related problems. Patient education and counseling. 2007 Mar 31;65(3):407-15.

60. Interian A, Martinez IE, Guarnaccia PJ, Vega WA, Escobar JI. A qualitative analysis of the perception of stigma among Latinos receiving antidepressants. Psychiatric Services. 2007 Dec;58(12):1591-4.

61. Campero L, Herrera C, Kendall T, Caballero M. Bridging the gap between antiretroviral access and adherence in Mexico. Qualitative Health Research. 2007 May;17(5):599-611.

62. Elliott RA, Ross-Degnan D, Adams AS, Safran DG, Soumerai SB. Strategies for coping in a complex world: adherence behavior among older adults with chronic illness. Journal of general internal medicine. 2007 Jun 1;22(6):805-10.

63. Bane C, Hughes CM, Cupples ME, McElnay JC. The journey to concordance for patients with hypertension: a qualitative study in primary care. Pharmacy world & science. 2007 Oct 1;29(5):534-40.

64. Sidat M, Fairley C, Grierson J. Experiences and perceptions of patients with 100% adherence to highly active antiretroviral therapy: a qualitative study. AIDS patient care and STDs. 2007 Jul 1;21(7):509-20.

65. Kagee A, Le Roux M, Dick J. Treatment adherence among primary care patients in a historically disadvantaged community in South Africa: A qualitative study. Journal of Health Psychology. 2007 May;12(3):444-60.

66. Piguet V, Cedraschi C, Dumont P, Desmeules J, Allaz AF, Dayer P. Patients' representations of antidepressants: a clue to nonadherence?. The Clinical journal of pain. 2007 Oct 1;23(8):669-75.

67. Orr A, Orr D, Willis S, Holmes M, Britton P. Patient perceptions of factors influencing adherence to medication following kidney transplant. Psychology, health & medicine. 2007 Aug 1;12(4):509-17.

68. Goff SL, Mazor KM, Meterko V, Dodd K, Sabin J. Patients’ beliefs and preferences regarding doctors’ medication recommendations. Journal of general internal medicine. 2008 Mar 1;23(3):236-41.

69. Unge C, Johansson A, Zachariah R, Some D, Van Engelgem IA, Ekstrom AM. Reasons for unsatisfactory acceptance of antiretroviral treatment in the urban Kibera slum, Kenya. AIDS care. 2008 Feb 1;20(2):146-9.

70. Brion JM, Menke EM. Perspectives regarding adherence to prescribed treatment in highly adherent HIV-infected gay men. Journal of the Association of Nurses in AIDS Care. 2008 Jun 30;19(3):181-91.

71. Wrubel J, Stumbo S, Johnson MO. Antiretroviral medication support practices among partners of men who have sex with men: A qualitative study. AIDS patient care and STDs. 2008 Nov 1;22(11):851-8.

72. Fongwa MN, Evangelista LS, Hays RD, Martins DS, Elashoff D, Cowan MJ, Morisky DE. Adherence treatment factors in hypertensive African American women. Vascular health and risk management. 2008 Apr 7;4(1).

73. Banbury P, Feenan K, Allcock N. Experiences of analgesic use in patients with low back pain. British Journal of Nursing. 2008 Oct 23;17(19).

74. Aspeling HE, Van Wyk NC. Factors associated with adherence to antiretroviral therapy for the treatment of HIV‐infected women attending an urban care facility. International journal of nursing practice. 2008 Feb 1;14(1):3-10.

75. Konkle-Parker DJ, Erlen JA, Dubbert PM. Barriers and facilitators to medication adherence in a southern minority population with HIV disease. Journal of the Association of Nurses in AIDS Care. 2008 Apr 30;19(2):98-104.

76. Elaine L, Papaioannou A, Dolovich L, Adachi J, Sawka AM, Burns S, Nair K, Pathak A. Patients’ adherence to osteoporosis therapy Exploring the perceptions of postmenopausal women. Canadian Family Physician. 2008 Mar 1;54(3):394-402.

77. Lehane E, McCarthy G, Collender V, Deasy A. Medication-taking for coronary artery disease—patients' perspectives. European Journal of Cardiovascular Nursing. 2008 Jun;7(2):133-9.

78. Miasso AI, Cassiani SH, Pedrão LJ. Bipolar affective disorder and medication therapy: identifying barriers. Revista latino-americana de enfermagem. 2008 Aug;16(4):739-45.

79. Merzel C, VanDevanter N, Irvine M. Adherence to antiretroviral therapy among older children and adolescents with HIV: a qualitative study of psychosocial contexts. AIDS patient care and STDs. 2008 Dec 1;22(12):977-87.

80. Lindberg M, Lindberg P. Overcoming obstacles for adherence to phosphate binding medication in dialysis patients: a qualitative study. Pharmacy World & Science. 2008 Oct 1;30(5):571-6.

81. Sanjobo N, Frich JC, Fretheim A. Barriers and facilitators to patients\'adherence to antiretroviral treatment in Zambia: a qualitative study. SAHARA-J: Journal of Social Aspects of HIV/AIDS. 2008;5(3):136-43.

82. Smith FJ, Taylor KM, Newbould J, Keady S. Medicines for chronic illness at school: experiences and concerns of young people and their parents. Journal of clinical pharmacy and therapeutics. 2008 Oct 1;33(5):537-44.

83. Sabin LL, Desilva MB, Hamer DH, Keyi X, Yue Y, Wen F, Tao L, Heggenhougen HK, Seton L, Wilson IB, Gill CJ. Barriers to adherence to antiretroviral medications among patients living with HIV in southern China: a qualitative study. AIDS care. 2008 Nov 1;20(10):1242-50.

84. Bokhour BG, Cohn ES, Cortés DE, Yinusa-Nyahkoon LS, Hook JM, Smith LA, Rand CS, Lieu TA. Patterns of concordance and non-concordance with clinician recommendations and parents’ explanatory models in children with asthma. Patient education and counseling. 2008 Mar 31;70(3):376-85.

85. Beusterien KM, Davis EA, Flood R, Howard K, Jordan J. HIV patient insight on adhering to medication: a qualitative analysis. AIDS care. 2008 Feb 1;20(2):244-52.

86. dosreis S, Mychailyszyn MP, Evans-Lacko SE, Beltran A, Riley AW, Myers MA. The meaning of attention-deficit/hyperactivity disorder medication and parents' initiation and continuity of treatment for their child. Journal of child and adolescent psychopharmacology. 2009 Aug 1;19(4):377-83.

87. Chong JJ, Davidsson A, Moles R, Saini B. What affects asthma medicine use in children? Australian asthma educator perspectives. Journal of Asthma. 2009 Jan 1;46(5):437-44.

88. Garavalia L, Garavalia B, Spertus JA, Decker C. Exploring patients’ reasons for discontinuance of heart medications. The Journal of cardiovascular nursing. 2009 Sep;24(5):371.

89. Granger BB, Sandelowski M, Tahshjain H, Swedberg K, Ekman I. A qualitative descriptive study of the work of adherence to a chronic heart failure regimen: patient and physician perspectives. Journal of Cardiovascular Nursing. 2009 Jul 1;24(4):308-15.

90. Gusdal AK, Obua C, Andualem T, Wahlström R, Tomson G, Peterson S, Ekström AM, Thorson A, Chalker J, Fochsen G, on behalf of the INRUD-IAA project. Voices on adherence to ART in Ethiopia and Uganda: a matter of choice or simply not an option?. AIDS care. 2009 Nov 1;21(11):1381-7.

91. Brinkman WB, Sherman SN, Zmitrovich AR, Visscher MO, Crosby LE, Phelan KJ, Donovan EF. Parental angst making and revisiting decisions about treatment of attention-deficit/hyperactivity disorder. Pediatrics. 2009 Aug 1;124(2):580-9.

92. Hansen DL, Holstein BE, Hansen EH. “I'd Rather Not Take it, But...”: Young Women's Perceptions of Medicines. Qualitative Health Research. 2009 Jun 1;19(6):829-39.

93. Haslbeck JW, Schaeffer D. Routines in medication management: the perspective of people with chronic conditions. Chronic Illness. 2009 Sep;5(3):184-96.

94. Hughes CM, Goldie R. I just take what I am given. Drugs & aging. 2009 Jun 1;26(6):505-17.

95. Vervoort SC, Grypdonck MH, De Grauwe A, Hoepelman AI, Borleffs JC. Adherence to HAART: processes explaining adherence behavior in acceptors and non-acceptors. AIDS care. 2009 Apr 1;21(4):431-8.

96. Vreeman RC, Nyandiko WM, Ayaya SO, Walumbe EG, Marrero DG, Inui TS. Factors sustaining pediatric adherence to antiretroviral therapy in western Kenya. Qualitative Health Research. 2009 Dec 1;19(12):1716-29.

97. Watt MH, Maman S, Earp JA, Eng E, Setel PW, Golin CE, Jacobson M. “It's all the time in my mind”: Facilitators of adherence to antiretroviral therapy in a Tanzanian setting. Social science & medicine. 2009 May 31;68(10):1793-800.

98. Williams AF, Manias E, Walker R. The role of irrational thought in medicine adherence: people with diabetic kidney disease. Journal of advanced nursing. 2009 Oct 1;65(10):2108-17.

99. Kelly J, D’Cruz G, Wright D. Patients with dysphagia: experiences of taking medication. Journal of advanced nursing. 2010 Jan 1;66(1):82-91.

100. Kourrouski MF, Lima RA. Treatment adherence: the experience of adolescents with HIV/AIDS. Revista latino-americana de enfermagem. 2009 Dec;17(6):947-52.

101. Lacey J, Cate H, Broadway DC. Barriers to adherence with glaucoma medications: a qualitative research study. Eye. 2009 Apr 1;23(4):924-32.

102. McMullen LM, Herman J. Women’s accounts of their decision to quit taking antidepressants. Qualitative Health Research. 2009 Nov;19(11):1569-79.

103. Stewart DC, Anthony GB, Chesson R. ‘It's not my job. I’m the patient not the doctor’: Patient perspectives on medicines management in the treatment of schizophrenia. Patient education and counseling. 2010 Feb 28;78(2):212-7.

104. Ruppar TM, Russell CL. Medication adherence in successful kidney transplant recipients. Progress in Transplantation. 2009 Jun 1;19(2):167-72.

105. Murray LK, Semrau K, McCurley E, Thea DM, Scott N, Mwiya M, Kankasa C, Bass J, Bolton P. Barriers to acceptance and adherence of antiretroviral therapy in urban Zambian women: a qualitative study. AIDS care. 2009 Jan 1;21(1):78-86.

106. Biadgilign S, Deribew A, Amberbir A, Deribe K. Barriers and facilitators to antiretroviral medication adherence among HIV-infected paediatric patients in Ethiopia: A qualitative study. SAHARA-J: Journal of Social Aspects of HIV/AIDS. 2009;6(4).

107. Stevens PE, Hildebrandt E. Pill taking from the perspective of HIV-infected women who are vulnerable to antiretroviral treatment failure. Qualitative Health Research. 2009 May;19(5):593-604.

108. Curioso WH, Kepka D, Cabello R, Segura P, Kurth AE. Understanding the facilitators and barriers of antiretroviral adherence in Peru: a qualitative study. BMC public health. 2010 Jan 13;10(1):13.

109. Guimarães C, Marra CA, Gill S, Meneilly G, Simpson S, Godoy AL. Exploring patients’ perceptions for insulin therapy in type 2 diabetes: a Brazilian and Canadian qualitative study. Patient preference and adherence. 2010;4:171.

110. Harrold LR, Mazor KM, Velten S, Ockene IS, Yood RA. Patients and providers view gout differently: a qualitative study. Chronic illness. 2010 Dec;6(4):263-71.

111. Hill-Smith I, Mathie E, Little P. Involving patients in decisions about preventive medication: a focus group study. Primary Care Cardiovascular Journal. 2010 Jan 1.

112. Howes F, Hansen E, Williams D, Nelson M. Barriers to diagnosing and managing hypertension: a qualitative study in Australian general practice. Australian family physician. 2010 Jul 1;39(7):511.

113. Lewis LM, Askie P, Randleman S, Shelton-Dunston B. Medication adherence beliefs of community-dwelling hypertensive African Americans. Journal of Cardiovascular Nursing. 2010 May 1;25(3):199-206.

114. Michaud PA, Suris JC, Thomas R, Gnehm HE, Cheseaux JJ. Coping with an HIV infection. A multicenter qualitative survey on HIV positive adolescents' perceptions of their disease, therapeutic adherence and treatment. Schweizerische medizinische Wochenschrift. 2010;140(17-18):247-53.

115. Mohammadpour A, Yekta ZP, Nikbakht Nasrabadi AR. HIV‐infected patients' adherence to highly active antiretroviral therapy: A phenomenological study. Nursing & health sciences. 2010 Dec 1;12(4):464-9.

116. Wang Y, Henning M. Bipolar disorder and medical adherence: A Chinese perspective. Asian journal of psychiatry. 2010 Mar 31;3(1):7-11.

117. Duxbury JA, Wright K, Bradley D, Barnes P. Administration of medication in the acute mental health ward: Perspective of nurses and patients. International Journal of Mental Health Nursing. 2010 Feb 1;19(1):53-61.

118. Wai KC, Elley CR, Nosa V, Kennelly J, Mabotuwana T, Warren J. Perspectives on adherence to blood pressure–lowering medications among samoan patients: qualitative interviews. Journal of primary health care. 2010 Sep 1;2(3):217-24.

119. Abrahams N, Jewkes R. Barriers to post exposure prophylaxis (PEP) completion after rape: a South African qualitative study. Culture, health & sexuality. 2010 Jun 1;12(5):471-84.

120. Bolster D, Manias E. Person-centred interactions between nurses and patients during medication activities in an acute hospital setting: qualitative observation and interview study. International journal of nursing studies. 2010 Feb 28;47(2):154-65.

121. Rifkin DE, Laws MB, Rao M, Balakrishnan VS, Sarnak MJ, Wilson IB. Medication adherence behavior and priorities among older adults with CKD: a semistructured interview study. American Journal of Kidney Diseases. 2010 Sep 30;56(3):439-46.

122. Matlock DD, Nowels CT, Bekelman DB. Patient perspectives on decision making in heart failure. Journal of cardiac failure. 2010 Oct 31;16(10):823-6.

123. Chambers JA, O’Carroll RE, Hamilton B, Whittaker J, Johnston M, Sudlow C, Dennis M. Adherence to medication in stroke survivors: a qualitative comparison of low and high adherers. British journal of health psychology. 2011 Sep 1;16(3):592-609.

124. Fredriksen-Goldsen KI, Shiu CS, Starks H, Chen WT, Simoni J, Kim HJ, Pearson C, Zhao H, Zhang F. “You must take the medications for you and for me”: family caregivers promoting HIV medication adherence in China. AIDS patient care and STDs. 2011 Dec 1;25(12):735-41.

125. Garavalia L, Ho PM, Garavalia B, Foody JM, Kruse H, Spertus JA, Decker C. Clinician–patient discord: Exploring differences in perspectives for discontinuing clopidogrel. European Journal of Cardiovascular Nursing. 2011 Mar;10(1):50-5.

126. Grant RW, Pabon-Nau L, Ross KM, Youatt EJ, Pandiscio JC, Park ER. Diabetes Oral Medication Initiation and Intensification Patient Views Compared With Current Treatment Guidelines. The Diabetes Educator. 2011;37(1):78-84.

127. Hommel KA, Odell S, Sander E, Baldassano RN, Barg FK. Treatment adherence in paediatric inflammatory bowel disease: perceptions from adolescent patients and their families. Health & social care in the community. 2011 Jan 1;19(1):80-8.

128. Iversen MD, Vora RR, Servi A, Solomon DH. Factors affecting adherence to osteoporosis medications: a focus group approach examining viewpoints of patients and providers. Journal of geriatric physical therapy. 2011 Apr;34(2):72.

129. Milder TY, Lipworth WL, Williams KM, Ritchie JE, Day RO. “It looks after me”: how older patients make decisions about analgesics for osteoarthritis. Arthritis care & research. 2011 Sep 1;63(9):1280-6.

130. Mills A, Lathlean J, Bressington D, Forrester A, Van Veenhuyzen W, Gray R. Prisoners' experiences of antipsychotic medication: influences on adherence. The Journal of Forensic Psychiatry & Psychology. 2011 Feb 1;22(1):110-25.

131. Toverud EL, Røise AK, Hogstad G, Wabø I. Norwegian patients on generic antihypertensive drugs: a qualitative study of their own experiences. European journal of clinical pharmacology. 2011 Jan 1;67(1):33-8.

132. Tranulis C, Goff D, Henderson DC, Freudenreich O. Becoming adherent to antipsychotics: a qualitative study of treatment-experienced schizophrenia patients. Psychiatric Services. 2011 Aug;62(8):888-92.

133. van Geffen EC, Hermsen JH, Heerdink ER, Egberts AC, Verbeek-Heida PM, van Hulten R. The decision to continue or discontinue treatment: experiences and beliefs of users of selective serotonin-reuptake inhibitors in the initial months—a qualitative study. Research in Social and Administrative Pharmacy. 2011 Jun 30;7(2):134-50.

134. Van Tam V, Pharris A, Thorson A, Alfven T, Larsson M. “It is not that I forget, it's just that I don't want other people to know”: barriers to and strategies for adherence to antiretroviral therapy among HIV patients in Northern Vietnam. AIDS care. 2011 Feb 1;23(2):139-45.

135. Wamboldt FS, Bender BG, Rankin AE. Adolescent decision-making about use of inhaled asthma controller medication: results from focus groups with participants from a prior longitudinal study. Journal of Asthma. 2011 Sep 1;48(7):741-50.

136. Watermeyer J. “Now here come the pills that are going to save your life”: pharmacists' discussions of antiretroviral drugs in a context of life and death. AIDS care. 2011 Jul 1;23(7):807-13.

137. Williams B, Amico KR, Konkle-Parker D. Qualitative assessment of barriers and facilitators to HIV treatment. The Journal of the Association of Nurses in AIDS Care: JANAC. 2011 Jul;22(4):307.

138. Awiti Ujiji O, Ekström AM, Ilako F, Indalo D, Wamalwa D, Rubenson B. Reasoning and deciding PMTCT-adherence during pregnancy among women living with HIV in Kenya. Culture, health & sexuality. 2011 Aug 1;13(7):829-40.

139. Axelsson M, Lötvall J, Lundgren J, Brink E. Motivational foci and asthma medication tactics directed towards a functional day. BMC public health. 2011 Oct 17;11(1):809.

140. Kranke DA, Floersch J, Kranke BO, Munson MR. A qualitative investigation of self-stigma among adolescents taking psychiatric medication. Psychiatric Services. 2011 Aug;62(8):893-9.

141. Klok T, Brand PL, Bomhof‐Roordink H, Duiverman EJ, Kaptein AA. Parental illness perceptions and medication perceptions in childhood asthma, a focus group study. Acta paediatrica. 2011 Feb 1;100(2):248-52.

142. Sale JE, Gignac MA, Hawker G, Frankel L, Beaton D, Bogoch E, Elliot-Gibson V. Decision to take osteoporosis medication in patients who have had a fracture and are'high'risk for future fracture: a qualitative study. BMC musculoskeletal disorders. 2011 May 9;12(1):92.

143. Salt E, Peden A. The complexity of the treatment: the decision-making process among women with rheumatoid arthritis. Qualitative Health Research. 2011 Feb;21(2):214-22.

144. Stumbo S, Wrubel J, Johnson MO. A qualitative study of HIV treatment adherence support from friends and family among same sex male couples. Psychology and education. 2011 Jul;2(4):318.

145. Penn C, Watermeyer J, Evans M. Why don’t patients take their drugs? The role of communication, context and culture in patient adherence and the work of the pharmacist in HIV/AIDS. Patient education and counseling. 2011 Jun 30;83(3):310-8.

146. Arrivillaga M, Ross M, Useche B, Springer A, Correa D. Applying an expanded social determinant approach to the concept of adherence to treatment: The case of Colombian women living with HIV/AIDS. Women's Health Issues. 2011 Apr 30;21(2):177-83.

147. Badahdah AM, Pedersen DE. “I want to stand on my own legs”: A qualitative study of antiretroviral therapy adherence among HIV-positive women in Egypt. AIDS care. 2011 Jun 1;23(6):700-4.

148. Ballantyne PJ, Mirza RM, Austin Z, Boon HS, Fisher JE. Becoming old as a ‘pharmaceutical person’: negotiation of health and medicines among ethnoculturally diverse older adults. Canadian Journal on Aging/La Revue canadienne du vieillissement. 2011 Jun 1;30(02):169-84.

149. Rust C, Davis C. Health literacy and medication adherence in underserved African-American breast cancer survivors: a qualitative study. Social work in health care. 2011 Oct 1;50(9):739-61.

150. Armitage G, Hodgson I, Wright J, Bailey K, Mkhwana E. Exploring the delivery of antiretroviral therapy for symptomatic HIV in Swaziland: threats to the successful treatment and safety of outpatients attending regional and district clinics. BMJ quality & safety. 2011 Jan 1;20(1):52-9.

151. Landier W, Hughes CB, Calvillo ER, Anderson NL, Briseño-Toomey D, Dominguez L, Martinez AM, Hanby C, Bhatia S. A grounded theory of the process of adherence to oral chemotherapy in Hispanic and caucasian children and adolescents with acute lymphoblastic leukemia. Journal of Pediatric Oncology Nursing. 2011 Jul 1;28(4):203-23.

152. Borgsteede SD, Westerman MJ, Kok IL, Meeuse JC, de Vries TP, Hugtenburg JG. Factors related to high and low levels of drug adherence according to patients with type 2 diabetes. International journal of clinical pharmacy. 2011 Oct 1;33(5):779.

153. Decker C, Garavalia L, Garavalia B, Simon T, Loeb M, Spertus JA, Daniel WC. Exploring barriers to optimal anticoagulation for atrial fibrillation: interviews with clinicians. Journal of multidisciplinary healthcare. 2012;5:129.

154. Coletti DJ, Pappadopulos E, Katsiotas NJ, Berest A, Jensen PS, Kafantaris V. Parent perspectives on the decision to initiate medication treatment of attention-deficit/hyperactivity disorder. Journal of child and adolescent psychopharmacology. 2012 Jun 1;22(3):226-37.

155. Cormier E. How Parents Make Decisions to Use Medication to Treat Their Child’s ADHD A Grounded Theory Study. Journal of the American Psychiatric Nurses Association. 2012 Nov 13:1078390312466918.

156. Granger BB, McBroom K, Bosworth HB, Hernandez A, Ekman I. The meanings associated with medicines in heart failure patients. European Journal of Cardiovascular Nursing. 2013 Jun;12(3):276-83.

157. Hon A. Factors influencing the adherence of antipsychotic medication (Aripiprazole) in first‐episode psychosis: findings from a grounded theory study. Journal of psychiatric and mental health nursing. 2012 May 1;19(4):354-61.

158. Brinkman WB, Sherman SN, Zmitrovich AR, Visscher MO, Crosby LE, Phelan KJ, Donovan EF. In their own words: adolescent views on ADHD and their evolving role managing medication. Academic pediatrics. 2012 Feb 29;12(1):53-61.

159. Jaarsma T, Nikolova-Simons M, van der Wal MH. Nurses' strategies to address self-care aspects related to medication adherence and symptom recognition in heart failure patients: An in-depth look. Heart & Lung: The Journal of Acute and Critical Care. 2012 Dec 31;41(6):583-93.

160. van den Boogaard J, Msoka E, Homfray M, Kibiki GS, Heldens JJ, Felling AJ, Aarnoutse RE. An exploration of patient perceptions of adherence to tuberculosis treatment in Tanzania. Qualitative health research. 2012 Jun 1;22(6):835-45.

161. Vilhelmsson A, Svensson T, Meeuwisse A, Carlsten A. Experiences from consumer reports on psychiatric adverse drug reactions with antidepressant medication: a qualitative study of reports to a consumer association. BMC Pharmacology and Toxicology. 2012 Dec 23;13(1):19.

162. Buus N, Johannessen H, Stage KB. Explanatory models of depression and treatment adherence to antidepressant medication: a qualitative interview study. International journal of nursing studies. 2012 Oct 31;49(10):1220-9.

163. Wasti SP, Simkhada P, Randall J, Freeman JV, Van Teijlingen E. Barriers to and facilitators of antiretroviral therapy adherence in Nepal: A qualitative study. Journal of health, population, and nutrition. 2012 Dec;30(4):410.

164. Watermeyer J, Penn C. “Only two months destroys everything”: a case study of communication about nonadherence to Antiretroviral therapy in a South African HIV pharmacy context. Health communication. 2012 Aug 1;27(6):602-11.

165. Watermeyer J. “This Clinic Is Number One” A Qualitative Study of Factors That Contribute Toward “Successful” Care at a South African Pediatric HIV/AIDS Clinic. Evaluation & the health professions. 2012 Sep;35(3):360-79.

166. Wendorf AR, Mosack KE. Navigating hazardous conditions: Understanding HIV medication adherence in the context of depression. Qualitative health research. 2013 Apr;23(4):541-54.

167. Widnes SF, Schjøtt J, Granas AG. Risk perception and medicines information needs in pregnant women with epilepsy–A qualitative study. Seizure. 2012 Oct 31;21(8):597-602.

168. Drey N, Mckeown E, Kelly D, Gould D. Adherence to antiparkinsonian medication: an in-depth qualitative study. International journal of nursing studies. 2012 Jul 31;49(7):863-71.

169. Arrivillaga M, Springer AE, Lopera M, Correa D, Useche B, Ross MW. HIV/AIDS treatment adherence in economically better off women in Colombia. AIDS care. 2012 Jul 1;24(7):929-35.

170. Besser SJ, Anderson JE, Weinman J. How do osteoporosis patients perceive their illness and treatment? Implications for clinical practice. Archives of osteoporosis. 2012 Dec 1;7(1-2):115-24.

171. Rushworth GF, Cunningham S, Mort A, Rudd I, Leslie SJ. Patient‐specific factors relating to medication adherence in a post‐percutaneous coronary intervention cohort. International Journal of Pharmacy Practice. 2012 Aug 1;20(4):226-37.

172. Kucukarslan SN, Lewis NJ, Shimp LA, Gaither CA, Lane DC, Baumer AL. Exploring patient experiences with prescription medicines to identify unmet patient needs: implications for research and practice. Research in Social and Administrative Pharmacy. 2012 Aug 31;8(4):321-32.

173. Lempp H, Hofmann D, Hatch SL, Scott DL. Patients’ views about treatment with combination therapy for Rheumatoid Arthritis: a comparative qualitative study. BMC musculoskeletal disorders. 2012 Oct 18;13(1):200.

174. Matovu SN, Hemmingsson H. Narratives of Ugandan women adhering to HIV/AIDS medication. Occupational therapy international. 2012 Dec 1;19(4):176-84.

175. Rogers B, Acton T. ‘I think we're all guinea pigs really’: a qualitative study of medication and borderline personality disorder. Journal of psychiatric and mental health nursing. 2012 May 1;19(4):341-7.

176. Brown TM, Siu K, Walker D, Pladevall-Vila M, Sander S, Mordin M. Development of a conceptual model of adherence to oral anticoagulants to reduce risk of stroke in patients with atrial fibrillation. Journal of Managed Care Pharmacy. 2012 Jun;18(5):351-62.

177. Bezreh T, Laws MB, Taubin T, Rifkin DE, Wilson IB. Challenges to physician-patient communication about medication use: a window into the skeptical patient’s world. Patient Prefer Adherence. 2012 Jan 1;6(1):11-8.

178. Bassett‐Clarke D, Krass I, Bajorek B. Ethnic differences of medicines‐taking in older adults: a cross cultural study in New Zealand. International Journal of Pharmacy Practice. 2012 Apr 1;20(2):90-8.

179. Lee YK, Lee PY, Ng CJ. A qualitative study on healthcare professionals’ perceived barriers to insulin initiation in a multi-ethnic population. BMC family practice. 2012 Jul 4;13(1):28.

180. Read U. “I want the one that will heal me completely so it won’t come back again”: The limits of antipsychotic medication in rural Ghana. Transcultural Psychiatry. 2012 Jul;49(3-4):438-60.

181. Santer M, Burgess H, Yardley L, Ersser S, Lewis-Jones S, Muller I, Hugh C, Little P. Experiences of carers managing childhood eczema and their views on its treatment: a qualitative study. Br J Gen Pract. 2012 Apr 1;62(597):e261-7.

182. Chang YT, Tao SG, Lu CL. Qualitative inquiry into motivators for maintaining medication adherence among Taiwanese with schizophrenia. International journal of mental health nursing. 2013 Jun 1;22(3):272-8.

183. Flynn SJ, Ameling JM, Hill-Briggs F, Wolff JL, Bone LR, Levine DM, Roter DL, Lewis-Boyer L, Fisher AR, Purnell L, Ephraim PL. Facilitators and barriers to hypertension self-management in urban African Americans: perspectives of patients and family members. Patient Prefer Adherence. 2013.

184. Griva K, Ng HJ, Loei J, Mooppil N, McBain H, Newman SP. Managing treatment for end-stage renal disease–a qualitative study exploring cultural perspectives on facilitators and barriers to treatment adherence. Psychology & Health. 2013 Jan 1;28(1):13-29.

185. Grover C, Goel N, Chugh K, Gaur SN, Armour C, Asperen PP, Moles RJ, Saini B. Medication use in Indian children with asthma: The user's perspective. Respirology. 2013 Jul 1;18(5):807-13.

186. Brion JM, Menke EM, Kimball C. Grief and HIV medication adherence: The work of transcending loss. Journal of Loss and Trauma. 2013 Nov 1;18(6):499-520.

187. Walstrom P, Operario D, Zlotnick C, Mutimura E, Benekigeri C, Cohen MH. ‘I think my future will be better than my past’: Examining support group influence on the mental health of HIV-infected Rwandan women. Global Public Health. 2013 Jan 1;8(1):90-105.

188. Waterman H, Brunton L, Fenerty C, Mottershead J, Richardson C, Spencer F. Adherence to ocular hypotensive therapy: patient health education needs and views on group education. Patient Prefer Adherence. 2013 Jan 1;7:55-63.

189. Stamer M, Schmacke N, Richter P. Noncompliance: A never-ending story. Understanding the perspective of patients with rheumatoid arthritis. InForum Qualitative Sozialforschung/Forum: Qualitative Social Research 2013 Jul 29 (Vol. 14, No. 3).

190. Teferra S, Hanlon C, Beyero T, Jacobsson L, Shibre T. Perspectives on reasons for non-adherence to medication in persons with schizophrenia in Ethiopia: a qualitative study of patients, caregivers and health workers. BMC psychiatry. 2013 Jun 17;13(1):168.

191. Murdoch J, Salter C, Cross J, Smith J, Poland F. Resisting medications: moral discourses and performances in illness narratives. Sociology of Health & Illness. 2013 Mar 1;35(3):449-64.

192. Stewart DW, DePue J, Rosen RK, Bereolos N, Goldstein MG, Tuitele J, Nu’usolia O, McGarvey ST. Medication-taking beliefs and diabetes in American Samoa: a qualitative inquiry. Translational behavioral medicine. 2013 Mar 1;3(1):30-8.
